# Mechanical Activation of Hypoxia-Inducible Factor 1α Drives Endothelial Dysfunction at Atheroprone Sites

**DOI:** 10.1161/ATVBAHA.117.309249

**Published:** 2017-10-25

**Authors:** Shuang Feng, Neil Bowden, Maria Fragiadaki, Celine Souilhol, Sarah Hsiao, Marwa Mahmoud, Scott Allen, Daniela Pirri, Blanca Tardajos Ayllon, Shamima Akhtar, A.A. Roger Thompson, Hanjoong Jo, Christian Weber, Victoria Ridger, Andreas Schober, Paul C. Evans

**Affiliations:** From the Department of Infection, Immunity, and Cardiovascular Disease, INSIGNEO Institute for In Silico Medicine, and the Bateson Centre (S.F., N.B., M.F., C.S., H.S., M.M., D.P., B.T.A., A.A.R.T., V.R., P.C.E.) and Sheffield Institute for Translational Neuroscience (S.A.), University of Sheffield, United Kingdom; Institute for Cardiovascular Prevention, Ludwig-Maximilians University of Munich and DZHK (German Centre for Cardiovascular Research), partner site Munich Heart Alliance, Germany (S.A., C.W., A.S.); and Wallace H. Coulter Department of Biomedical Engineering, Georgia Institute of Technology and Emory University, Atlanta (H.J.).

**Keywords:** apolipoproteins E, atherosclerosis, endothelial cells, glycolysis, hypoxia-inducible factor 1

## Abstract

**Objective—:**

Atherosclerosis develops near branches and bends of arteries that are exposed to low shear stress (mechanical drag). These sites are characterized by excessive endothelial cell (EC) proliferation and inflammation that promote lesion initiation. The transcription factor HIF1α (hypoxia-inducible factor 1α) is canonically activated by hypoxia and has a role in plaque neovascularization. We studied the influence of shear stress on HIF1α activation and the contribution of this noncanonical pathway to lesion initiation.

**Approach and Results—:**

Quantitative polymerase chain reaction and en face staining revealed that HIF1α was expressed preferentially at low shear stress regions of porcine and murine arteries. Low shear stress induced HIF1α in cultured EC in the presence of atmospheric oxygen. The mechanism involves the transcription factor nuclear factor-κB that induced HIF1α transcripts and induction of the deubiquitinating enzyme Cezanne that stabilized HIF1α protein. Gene silencing revealed that HIF1α enhanced proliferation and inflammatory activation in EC exposed to low shear stress via induction of glycolysis enzymes. We validated this observation by imposing low shear stress in murine carotid arteries (partial ligation) that upregulated the expression of HIF1α, glycolysis enzymes, and inflammatory genes and enhanced EC proliferation. EC-specific genetic deletion of HIF1α in hypercholesterolemic apolipoprotein E–defecient mice reduced inflammation and endothelial proliferation in partially ligated arteries, indicating that HIF1α drives inflammation and vascular dysfunction at low shear stress regions.

**Conclusions—:**

Mechanical low shear stress activates HIF1α at atheroprone regions of arteries via nuclear factor-κB and Cezanne. HIF1α promotes atherosclerosis initiation at these sites by inducing excessive EC proliferation and inflammation via the induction of glycolysis enzymes.

## Abstract 2

Supplemental Digital Content is available in the text.

Although it is associated with risk factors that act systemically (eg, hypercholesterolemia, smoking, age), atherosclerosis is a focal disease that develops preferentially near branches and bends of arteries.^1^ The physiology of endothelial cells (EC) varies considerably according to their location in the arterial tree. EC at regions that are predisposed to lesion formation (atheroprone) are characterized by relatively high rates of proliferation^2–4^ and proinflammatory activation.^5–9^ These features, which we refer to as endothelial dysfunction, promote early atherogenesis by increasing the accessibility of the vessel wall to leukocytes and its permeability to cholesterol-rich lipoproteins^10^ that are key drivers of atherogenesis. By contrast, EC at atheroprotected sites remain quiescent and are resistant to inflammatory activation.^1–9^ The spatial localization of EC phenotypes and atherosclerosis is tightly linked to local hemodynamics. Blood flow generates a frictional force at the endothelial surface called wall shear stress that varies in magnitude and direction according to vascular anatomy. Atheroprotected regions of arteries with relatively uniform geometry are exposed to high time-averaged shear stress that is unidirectional, whereas atheroprone sites near branches and bends are exposed to complex flow patterns generating low time-averaged shear stress that varies in direction (eg, oscillatory bidirectional and biaxial flow).^9,11^ The application of flow to cultured cells has revealed a causal relationship between flow and EC physiology. Atheroprotective flow patterns induce numerous coding and noncoding RNAs that induce quiescence and reduce inflammation, whereas atheroprone flow activates multiple signaling pathways and transcription factors that promote EC dysfunction and inflammation.^12^

**See accompanying editorial on page 1987**

The transcription factor HIF1α (hypoxia-inducible factor 1α) is a central regulator of cellular responses to hypoxia. In cells exposed to physiological levels of oxygen (normoxia), HIF1α is modified with hydroxyl groups by prolyl hydroxylase domain (PHD) enzymes, thus targeting it for rapid ubiquitination and degradation.^13–16^ However, this oxygen-dependent process is inactivated in conditions of hypoxia, leading to HIF1α accumulation. HIF1α plays an essential role during angiogenesis by inducing vascular endothelial growth factor and other growth factors, thereby enhancing perfusion of ischemic tissues to restore oxygenation.^17^ Of note, this process also requires HIF1α-dependent induction of multiple glycolytic enzymes,^18^ thus causing a metabolic switch that allows ATP and macromolecules to be generated by glycolysis under anaerobic conditions.^19–21^ HIF1α is expressed in advanced atherosclerotic plaques,^22^ and a recent study demonstrated that genetic deletion of HIF1α from EC reduced experimental atherosclerosis in a murine model.^23^ However, the potential role of HIF1α in focal EC dysfunction and inflammation linked to early atherogenesis has not been studied.

Here, we show for the first time that HIF1α can be activated in EC by mechanical low shear stress, leading to enrichment of HIF1α expression at atheroprone regions of arteries. HIF1α is upregulated via a dual mechanism involving transcriptional activation by nuclear factor (NF)-κB and stabilization via the deubiquitinating enzyme Cezanne. At a functional level, we demonstrate that HIF1α drives atherogenic processes at predilection sites by enhancing inflammation and inducing excessive EC proliferation via upregulation of glycolysis enzymes. Thus, mechanical activation of HIF1α is a novel mechanism for the focal induction of atherosclerosis. This pathway could potentially provide a novel target to prevent or treat early atherosclerosis.

## Materials and Methods

Materials and Methods are available in the online-only Data Supplement.

### Animals

EC from high (outer) or low (inner curvature) shear stress regions of porcine aortae were isolated.^24^ HIF1α was deleted from EC of apolipoprotein E–defecient (ApoE^−/−^) mice (called HIF1α^EC-cKO^) using tamoxifen,^23^ and the left carotid artery was partially ligated^25^ before high-fat feeding. The expression of specific proteins was assessed by en face staining^3,7,8,24,26^ or by immunostaining of carotid artery cross-sections.^23^

### Cultured EC

Human umbilical vein EC were transfected with small interfering RNA^24,26^ or with expression vectors containing IκBα (nuclear factor of kappa light polypeptide gene enhancer in B-cells inhibitor, alpha; pCMV-IκBα, GFP [green fluorescent protein]-Cezanne, or GFP-Cezanne Cys/Ser [catalytically inactive]).^27^ Human umbilical vein EC and human coronary artery EC were exposed to flow using orbital shaking or an Ibidi parallel plate system.^24,26,28^ Quantitative reverse transcription polymerase chain reaction (qRT-PCR) was performed using gene-specific primers (Table I in the online-only Data Supplement), and immunofluorescent staining, Western blotting, and chromatin immunoprecipitation were performed with specific primary antibodies (Table II in the online-only Data Supplement). Glycolysis was monitored using the Seahorse system.^29^

## Results

### HIF1α and Glycolysis Enzymes Are Expressed at Atheroprone Sites

**Figure 1.**
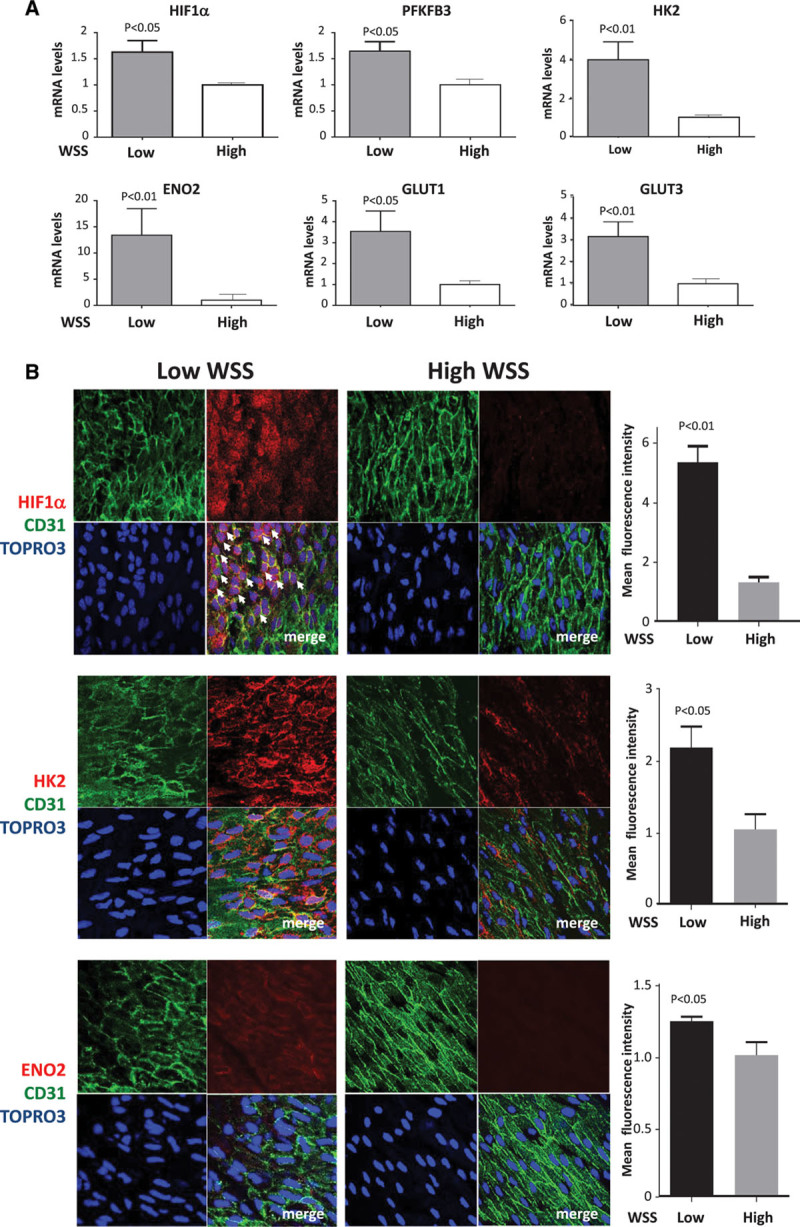
Enrichment of HIF1α (hypoxia-inducible factor 1α) and glycolytic enzymes at atheroprone sites. The expression of HIF1α and several of its target genes was quantified at low wall shear stress (WSS; inner curvature) and high WSS (outer curvature) regions of the porcine aorta by quantitative reverse transcription polymerase chain reaction (**A**) and en face staining of the mouse aorta (**B**). Mean levels±SEM are shown for n=5 pigs and n=3 mice. In representative images, endothelial cells were identified by costaining with anti-CD31 antibodies conjugated to fluorescein isothiocyanate (green). Cell nuclei were identified using TOPRO3 (blue). Arrows indicate nuclear HIF1α. Differences between means were analyzed using a paired *t* test. ENO2 indicates enolase 2; GLUT, glucose transporter; HK2, hexokinase 2; and PFKFB3, 6-phosphofructo-2-kinase/fructose-2,6-biphosphatase 3.

A recent study from our laboratory revealed >800 transcripts that were differentially expressed between EC at atheroprone (low shear stress; inner curvature of arch) and atheroprotected (high shear stress; outer curvature) regions of the porcine aorta.^24^ It suggested that HIF1α and several of its target genes were upregulated at the atheroprone site. To validate this observation, we performed qPCR studies of an independent cohort of pigs and demonstrated that EC isolated from the low shear stress region had enriched expression of HIF1α and several downstream targets, including the glycolysis regulator 6-phosphofructo-2-kinase/fructose-2,6-biphosphatase 3 (PFKFB3), glycolysis enzymes (hexokinase 2 [HK2], enolase 2), and glucose transporters (glucose transporter 1, glucose transporter 3; Figure 1A).

We also performed en face staining of the murine aortic endothelium to quantify the expression of HIF1α at sites that are known to be exposed to low (inner curvature of arch) or high (curvature) shear stress.^11^ It demonstrated that HIF1α protein was expressed at higher levels at a low shear compared with a high shear stress site (Figure 1B, top). Tiling of multiple fields of view revealed a sharp delineation in HIF1α expression, which was observed in EC exposed to low shear stress (note nonaligned nuclei) but not in EC exposed to high shear stress (aligned nuclei; Figure I in the online-only Data Supplement). It was concluded that HIF1α was active at the low shear region as a portion of the cellular pool localized to the nucleus (Figure 1B, top, arrows); in addition, the expression of HK2 and enolase 2 target molecules was also enriched at the low shear site (Figure 1B, center and bottom). The influence of atherogenesis on HIF1α expression was studied using ApoE^−/−^ mice exposed to a high-fat diet for 6 weeks. En face staining revealed that HIF1α was expressed in EC overlying plaques and that the level of expression at the low shear region was similar in wild-type and ApoE^−/−^ mice (Figure II in the online-only Data Supplement). Thus, we conclude that HIF1α and downstream glycolysis genes are expressed preferentially at low shear atheroprone sites and that HIF1α expression is maintained during early atherogenesis.

### Low Shear Stress Induces HIF1α in Conditions of Atmospheric Oxygen

**Figure 2.**
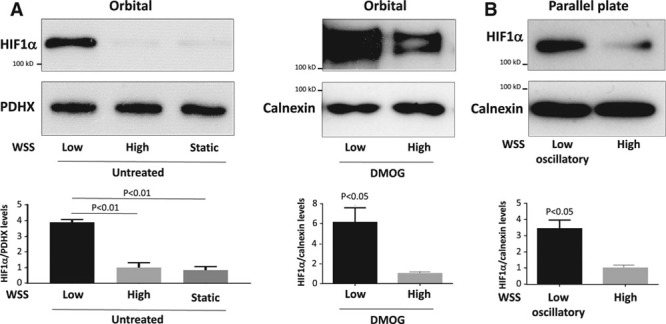
Low shear stress induced HIF1α (hypoxia-inducible factor 1α). **A**, Human umbilical vein endothelial cells (HUVEC) were exposed to orbital flow to generate low (5 dyn/cm^2^) or high (11 dyn/cm^2^) wall shear stress (WSS) or were maintained under static conditions. After 72 h, they were exposed to dimethyloxalylglycine (DMOG) for 4 h or remained untreated. **B**, Alternatively, HUVEC were exposed to high (13 dyn/cm^2^) or low oscillatory (4 dyn/cm^2^; 0.5 Hz) WSS for 72 h using a parallel plate system. **A** and **B**, The expression levels of HIF1α were assessed by Western blotting using specific antibodies, and anti-Calnexin or anti-PDHX (pyruvate dehydrogenase complex component X) antibodies were used to control for total protein levels. Representative blots are shown. Bands were quantified by densitometry. Data were pooled from 3 independent experiments, and mean HIF1α expression±SEM is shown. Differences between means were analyzed using a paired *t* test or 1-way ANOVA with the Bonferroni correction for multiple pairwise comparisons.

Given its expression at atheroprone sites, we hypothesized that HIF1α is regulated by shear stress. In preliminary studies, we validated the detection of HIF1α by Western blotting by demonstrating that anti-HIF1α antibodies recognize a single band (at ≈120 kDa) in cells treated with the PHD inhibitor dimethyloxalylglycine and that this band was suppressed by small interfering RNA sequences designed to target HIF1α (with no effect on HIF2α; Figure III in the online-only Data Supplement). The potential relationship between shear stress and HIF1α was investigated using cultured EC exposed to flow in the presence of atmospheric oxygen. Two complimentary systems were used: an orbital system that generates regions of lower shear stress (5 dyn/cm^2^) with variation in direction at the center and higher unidirectional shear stress (11 dyn/cm^2^) at the periphery^28^ and a parallel plate system that was used to generate unidirectional shear stress of 4 or 13 dyn/cm^2^. Although these shear stress magnitudes are within the physiological range, they are referred to as low (4–5 dyn/cm^2^) and high (11–13 dyn/cm^2^) shear stress, respectively, for the sake of brevity. We have previously validated both the orbital and parallel plate systems; for example, high shear stress reduces apoptosis by inducing antiapoptotic genes.^24^ First, we demonstrated using the orbital system that HIF1α expression was elevated in EC exposed to low shear conditions compared with cells exposed to high shear or static conditions (Figure 2A, left). Similarly, exposure of EC to flow using a parallel plate apparatus revealed enhanced expression of HIF1α in cells exposed to low oscillatory compared with high uniform shear stress (Figure 2B). We confirmed by piminidazole staining that these flow conditions did not induce hypoxia (Figure IV in the online-only Data Supplement). However, the induction of HIF1α in response to dimethyloxalylglycine (a hypoxia mimic) was elevated in EC exposed to low shear compared with cells exposed to high shear (Figure 2A, right). Thus, it was concluded that low shear stress induces HIF1α expression and also primes EC for enhanced HIF1α activation in response to hypoxic signaling.

### NF-κB and Cezanne-Dependent Mechanism Induce and Stabilize HIF1α Under Low Shear Stress

**Figure 3.**
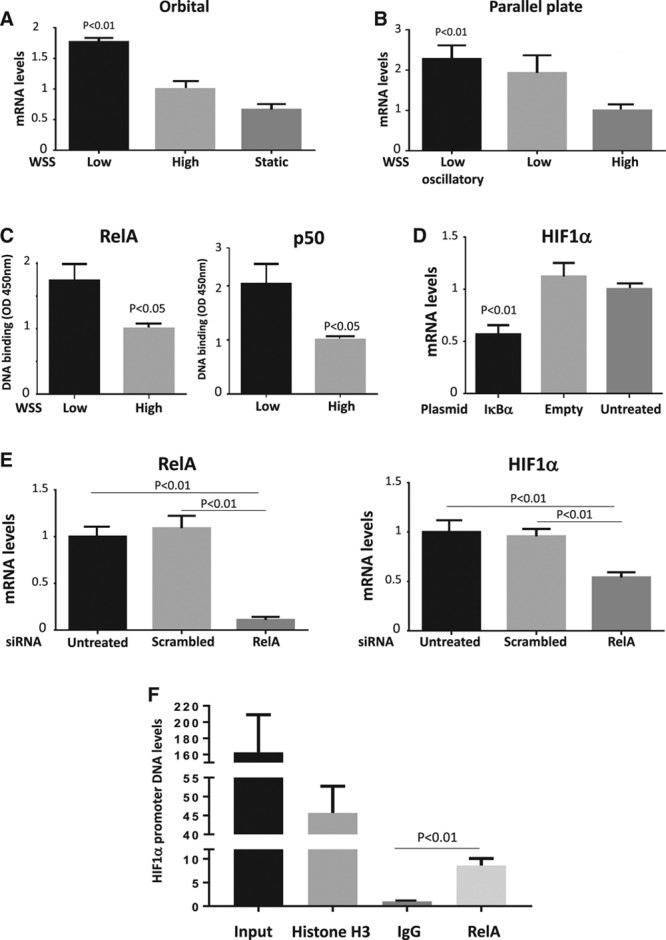
Nuclear factor-κB induced HIF1α (hypoxia-inducible factor 1α) mRNA in response to low shear stress. Human umbilical vein endothelial cells (HUVEC) were exposed to low or high wall shear stress (WSS) for 72 h using an orbital (5 vs 11 dyn/cm^2^; **A**) or parallel plate (4 vs 13 dyn/cm^2^; **B**) system. **A** and **B**, The expression levels of HIF1α mRNA were assessed by quantitative reverse transcription polymerase chain reaction (qRT-PCR). Mean HIF1α expression±SEM is shown. **C**, HUVEC were exposed to orbital flow to generate low or high WSS. After 72 h, the activity of RelA and p50 was assessed by DNA-binding enzyme-linked immunosorbent assay. Mean optical density (OD) 450 nm values±SEM are shown. **D**, HUVEC were transfected using pCMV-IκBα or with an empty plasmid or remained untreated as a control. **E**, HUVEC were transfected with small interfering RNA (siRNA) targeting RelA or with scrambled sequences. **D** and **E**, Cells were exposed to low WSS using the orbital system. After 72 h, the expression levels of HIF1α or RelA mRNA were assessed by qRT-PCR. Mean expression±SEM is shown. **F**, Nuclear lysates prepared from HUVECs exposed to orbital flow for 72 h were incubated with anti-RelA, antihistone H3, or irrelevant control antibodies before precipitation. The levels of HIF1α promoter DNA in precipitates and in nonprecipitated lysates (input) were assessed by comparative real-time PCR. Mean levels (SEM) are shown. **A**–**F**, Data were pooled from 3 independent experiments. Differences between means were analyzed using a 1-way ANOVA with the Bonferroni correction for multiple pairwise comparisons (**A**, **B**, **D**, **E**, **F**) or a paired *t* test (**C**).

The mechanism linking low shear stress to HIF1α upregulation was investigated. qRT-PCR revealed that HIF1α mRNA levels were significantly elevated in human umbilical vein EC exposed to low or low oscillatory shear stress using the orbital (Figure 3A) or parallel plate (Figure 3B) systems compared with cells exposed to high shear stress or static conditions, indicating that low shear stress induces HIF1α at the transcript level. Similarly, HIF1α mRNA levels were elevated in human coronary artery EC exposed to low shear stress compared with cells exposed to high shear stress or static conditions (Figure V in the online-only Data Supplement). We hypothesized that flow regulates HIF1α mRNA via NF-κB, a transcription factor that can induce HIF1α in response to inflammatory signaling.^30,31^ In support of this, DNA-binding enzyme-linked immunosorbent assay revealed that low shear stress activated RelA and p50 subunits in cultured EC (Figure 3C). A direct link between NF-κB and HIF1α expression was established using an expression plasmid containing IκBα, which inhibited NF-κB activity in transfected EC (Figure VI in the online-only Data Supplement). Overexpression of IκBα reduced HIF1α expression in EC exposed to low shear stress, whereas an empty control plasmid had no effect (Figure 3D), indicating that NF-κB positively regulated HIF1α under low shear conditions. Similarly, silencing of RelA NF-κB subunits reduced HIF1α expression in EC exposed to low shear (Figure 3E). Finally, chromatin immunoprecipitation studies demonstrated that HIF1α promoter sequences coprecipitated with antihistone H3 antibodies (positive control) and anti-RelA antibodies but did not coprecipitate with control IgG in EC exposed to orbital flow (Figure 3F), indicating that RelA interacts with the HIF1α promoter under low shear stress conditions. Collectively, these observations indicate that low shear stress induces HIF1α mRNA via an NF-κB–dependent process.

**Figure 4.**
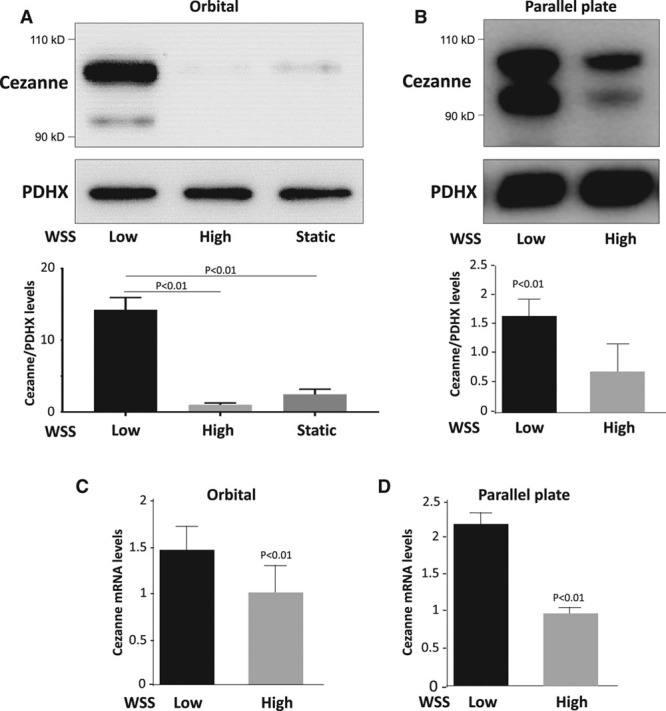
Low shear stress induced Cezanne. **A** and **C**, Human umbilical vein endothelial cells (HUVEC) were exposed to orbital flow to generate low (5 dyn/cm^2^) or high (11 dyn/cm^2^) wall shear stress (WSS) or were maintained under static conditions. **B** and **D**, Alternatively, HUVEC were exposed to high (13 dyn/cm^2^) or low (4 dyn/cm^2^) WSS for 72 h using a parallel plate system. **A** and **B**, The expression levels of Cezanne were assessed by Western blotting using specific antibodies, and anti-PDHX (pyruvate dehydrogenase complex component X) antibodies were used to control for total protein levels. Representative blots are shown. Bands were quantified by densitometry. Data were pooled from 3 independent experiments, and mean Cezanne expression±SEM is shown. **C** and **D**, The expression levels of Cezanne mRNA were assessed by quantitative reverse transcription polymerase chain reaction. Data were pooled from 3 independent experiments, and mean expression±SEM is shown. Differences between means were analyzed using a 1-way ANOVA with the Bonferroni correction for multiple pairwise comparisons (**A**) or a paired *t* test (**B**–**D**).

**Figure 5.**
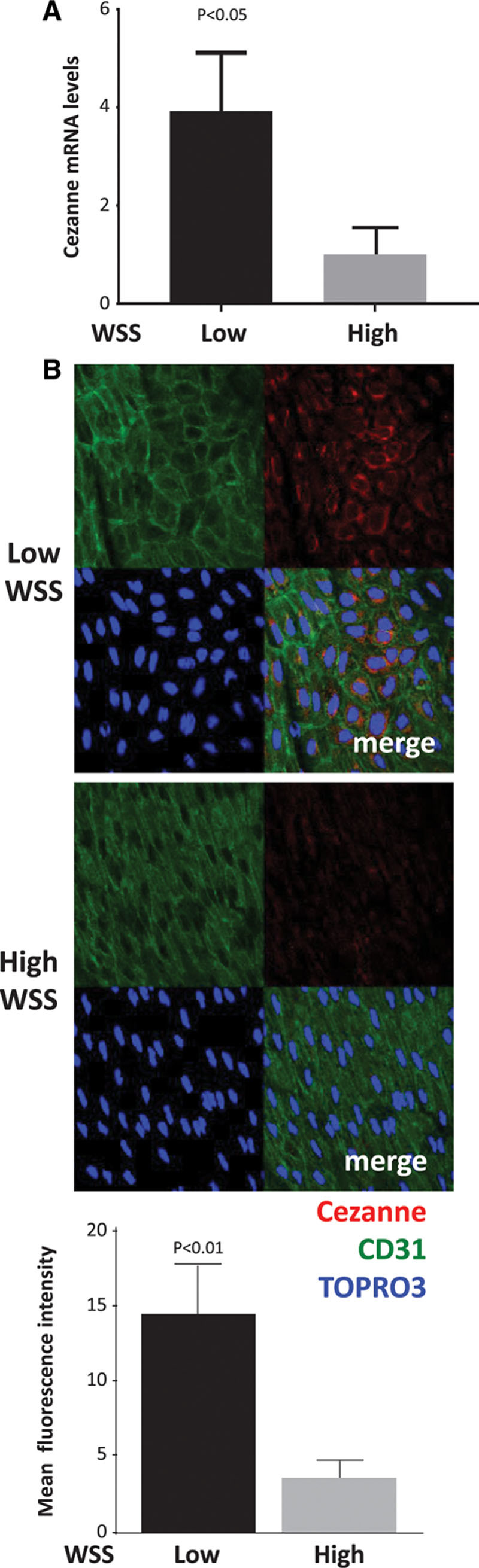
Enrichment of Cezanne at an atheroprone site. The expression of Cezanne was quantified at low wall shear stress (WSS; inner curvature) and high WSS (outer curvature) regions of the porcine aorta by quantitative reverse transcription polymerase chain reaction (**A**) and en face staining of the mouse aorta (**B**). Mean levels±SEM are shown for n=7 pigs and n=4 mice. In representative images, endothelial cells were identified by costaining with anti-CD31 antibodies conjugated to fluorescein isothiocyanate (green). Cell nuclei were identified using TOPRO3 (blue). Differences between means were analyzed using a paired *t* test.

We reasoned that low shear stress must also prevent HIF1α degradation to enhance its expression at the protein level. To identify the mechanism, we examined the effects of flow on the expression of PHD proteins and Von Hippel Lindau (VHL) E3 ubiquitin ligase which destabilize HIF1α by modifying it with hydroxyl groups and ubiquitin, respectively.^13–15^ Western blotting revealed that PHD1, PHD3, and VHL were not suppressed by low shear stress (data not shown), indicating that HIF1α accumulation under these conditions is not mediated by reduced hydroxylation or ubiquitination. However, HIF1α can be stabilized by the deubiquitinating enzyme Cezanne that rescues it from degradation.^32^ The expression of Cezanne in cultured EC exposed to flow was studied by Western blotting. Antibodies against the N-terminal portion generated bands at ≈95 and ≈105 kDa (Figure 4), which were confirmed to result from Cezanne expression by gene silencing (Figure VII in the online-only Data Supplement). It was concluded that Cezanne is expressed at significantly higher levels in EC exposed to low shear stress compared with cells exposed to high shear stress or static conditions (Figure 4A and 4B). Similarly, qRT-PCR demonstrated that Cezanne mRNA levels were enhanced under low compared with high shear stress (Figure 4C and 4D). These observations were validated by qRT-PCR analysis of EC isolated from regions of the porcine aortic arch (Figure 5A) and en face staining of the murine aortic endothelium (Figure 5B; Figure I in the online-only Data Supplement), which demonstrated that Cezanne protein was expressed at higher levels at low shear compared with high shear stress sites.

**Figure 6.**
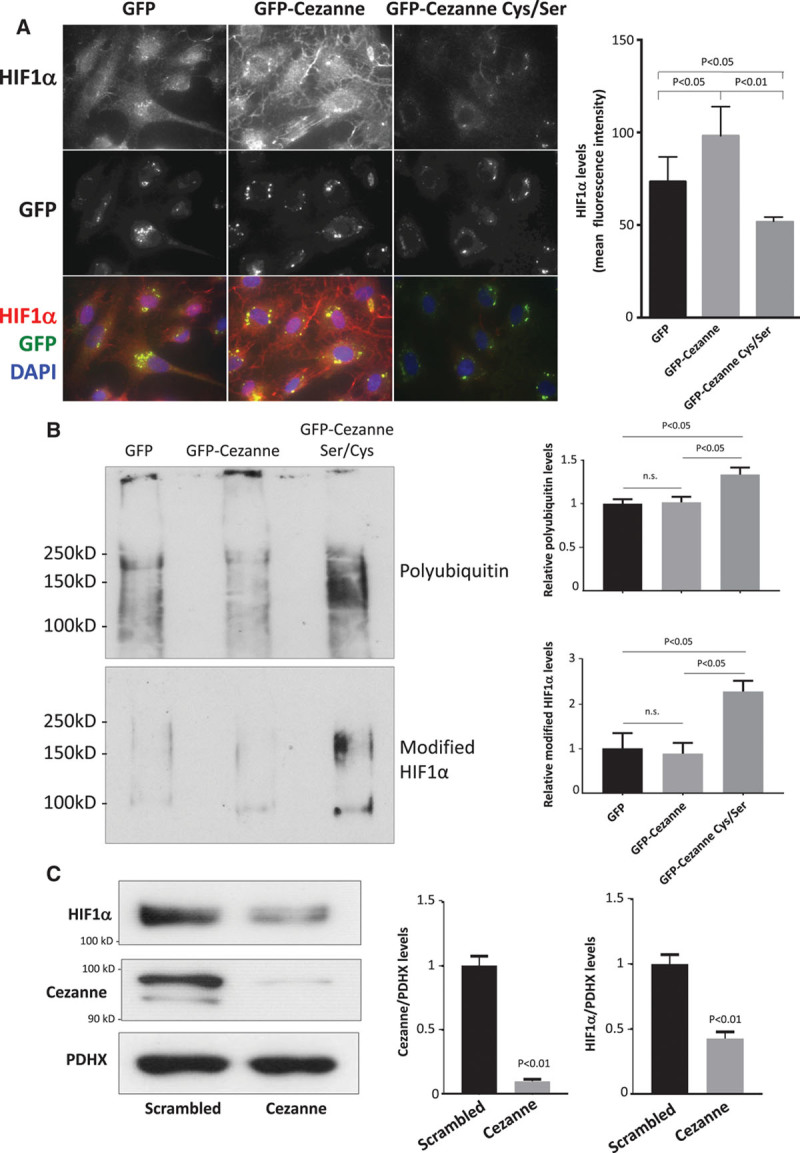
Cezanne stabilizes HIF1α (hypoxia-inducible factor 1α) expression under low shear stress conditions via deubiquitination. **A** and **B**, Human umbilical vein endothelial cells (HUVEC) were transfected using pGFP-Cezanne, pGFP-Cezanne Cys/Ser, or with pGFP alone as a control. Cells were subsequently exposed to low wall shear stress (WSS; 5 dyn/cm^2^) using the orbital system for 72 h. **A**, The expression levels of HIF1α were assessed by immunofluorescent staining. Representative images and quantification of HIF1α expression (mean±SEM) are shown. **B**, Cells were treated with MG132 (proteasome inhibitor; 50 μmol/L) and bafilomycin (lysosome inhibitor; 100 nmol/L) for the final 4 h of orbiting. HIF1α immunoprecipitates were tested by Western blotting using antiubiquitin (**top**) or anti-HIF1α (**bottom**) antibodies. Representative blots are shown. Bands were quantified by densitometry. Data were pooled from 3 independent experiments, and mean levels±SEM are shown. **C**, HUVEC were transfected with scrambled sequences or Cezanne siRNA and incubated for 24 h. Cells were exposed for 72 h to low WSS (5 dyn/cm^2^) using the orbital system. The expression levels of HIF1α and Cezanne were assessed by Western blotting using specific antibodies, and anti-PDHX (pyruvate dehydrogenase complex component X) antibodies were used to control for total protein levels. Representative blots are shown. Bands were quantified by densitometry. Data were pooled from 3 independent experiments, and mean levels±SEM are shown. **A** and **C**, Differences between means were analyzed using 1-way ANOVA with the Bonferroni correction for multiple pairwise comparisons (**A** and **B**) or a paired *t* test (**C**). DAPI indicates 4′,6-diamidino-2-phenylindole.

To assess whether Cezanne is responsible for stabilizing HIF1α under low shear stress, we transfected cells with a dominant negative form that is mutated at the catalytic cysteine (GFP-Cezanne Cys/Ser). Immunofluorescent staining revealed that the expression of HIF1α was reduced by expression of GFP-Cezanne Cys/Ser and increased by GFP-Cezanne (wild type) compared with control GFP-transfected cells (Figure 6A), indicating that Cezanne activity is required to enhance HIF1α protein expression under low shear stress conditions. The effects of Cezanne on HIF1α ubiquitination were assessed by Western blotting of HIF1α immunoprecipitates generated from EC exposed to orbital flow. The abundance of high molecular weight forms of HIF1α and coprecipitating polyubiquitin was enhanced by expression of GFP-Cezanne Cys/Ser compared with expression of GFP-Cezanne or GFP alone (Figure 6B). These data are consistent with the hypothesis that Cezanne can target HIF1α for deubiquitination in EC exposed to low shear stress. Consistent with these observations, silencing of Cezanne significantly reduced HIF1α expression at the protein level (Figure 6C) but not at the mRNA level (Figure VIII in the online-only Data Supplement) in EC exposed to low shear stress. In conclusion, HIF1α is upregulated by low shear stress via dual processes that induce it at a transcriptional level via NF-κB and enhance it at the protein level via Cezanne.

### HIF1α Promotes Glycolysis Under Low Shear Conditions

**Figure 7.**
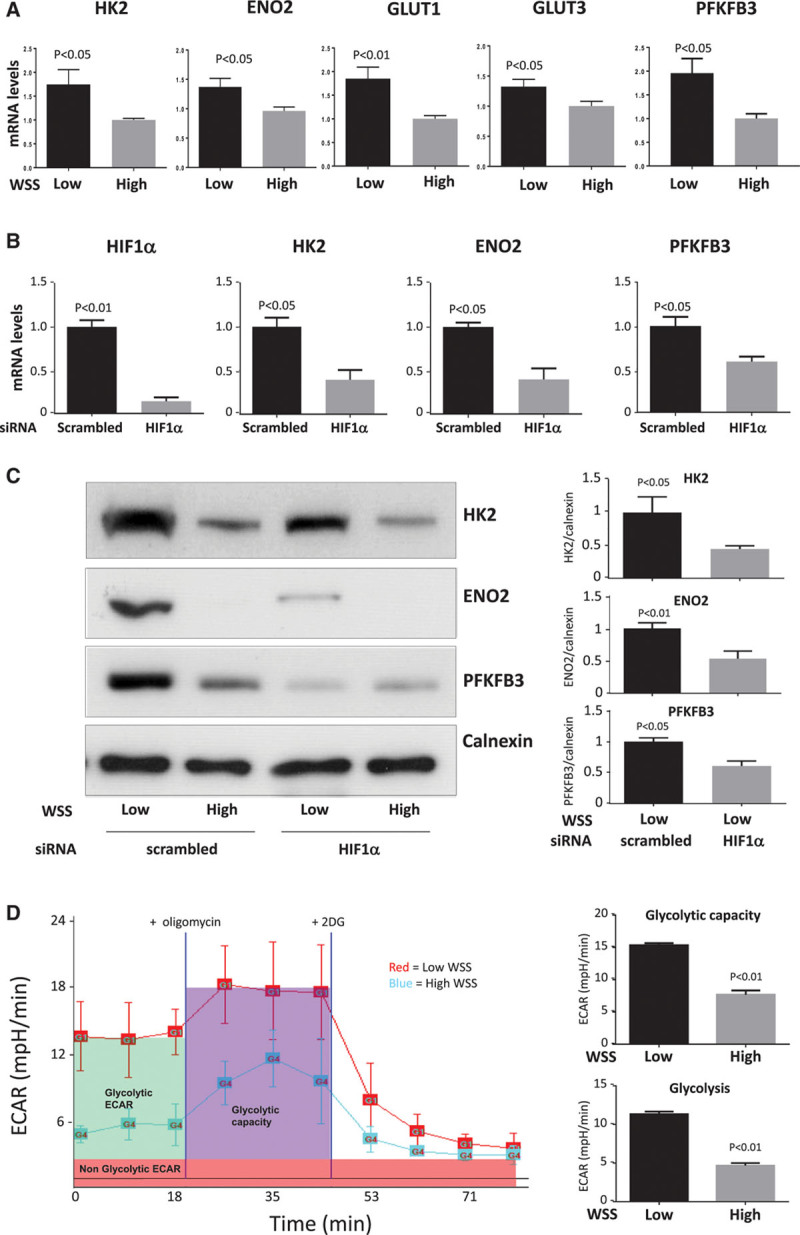
Low shear stress enhanced glycolysis via HIF1α (hypoxia-inducible factor 1α)-dependent induction of glycolytic enzymes. **A**, Human umbilical vein endothelial cells (HUVEC) were exposed to orbital flow to generate low (5 dyn/cm^2^) or high (11 dyn/cm^2^) wall shear stress (WSS). After 72 h, the expression levels of hexokinase 2 (HK2), enolase 2 (ENO2), glucose transporter (GLUT) 1, GLUT3, and PFKFB3 mRNA were assessed by quantitative reverse transcription polymerase chain reaction (qRT-PCR). **B** and **C**, HUVEC were transfected with small interfering RNA (siRNA) targeting HIF1α or with scrambled sequences. Cells were subsequently exposed to orbital flow to generate low WSS for 72 h. **B**, The expression levels of HIF1α, HK2, ENO2, and 6-phosphofructo-2-kinase/fructose-2,6-biphosphatase 3 (PFKFB3) mRNA were assessed by qRT-PCR. **C**, The expression levels of HK2, ENO2, PFKFB3, and Calnexin were assessed by Western blotting using specific antibodies. Representative blots are shown. Bands in low WSS samples were quantified by densitometry. **D**, Glycolytic bioenergetics profile of HUVECs subjected to low (red trace) or high (blue trace) WSS. HUVEC were exposed to low or high WSS for 72 h using an orbital system. Basal glycolytic extracellular acidification rate (ECAR; green) was measured before addition of oligomycin to assess glycolytic capacity (purple) and subsequently 2 deoxyglucose (2DG) to assess nonglycolytic ECAR (red). ECAR traces are representative of those generated in 3 independent experiments and are displayed as the mean of 3 wells±SEM (**left**). Mean glycolytic ECAR and glycolytic capacity±SEM are shown (**right**). **A**–**D**, Data were generated from 3 independent experiments, and differences between means were analyzed using a paired *t* test.

HIF1α drives angiogenesis by inducing a glycolytic switch,^17–21^ and we wondered whether this process can also be induced by low shear stress. qRT-PCR and Western blotting revealed that several glycolysis regulators, including HK2, enolase 2, and PFKFB3, were induced in cultured EC by the application of low shear stress (Figure 7A and 7C). Their expression was reduced by silencing of HIF1α (Figure 7B and 7C) or by silencing of Cezanne (Figure VIII in the online-only Data Supplement) and enhanced in cells exposed to dimethyloxalylglycine (Figure IX in the online-only Data Supplement), indicating that Cezanne-HIF1α signaling activates glycolysis genes in EC exposed to low shear stress.

To study the influence of shear stress on glycolysis directly, we applied flow to cultured EC for 72 hours and then monitored extracellular acidification rate using a Seahorse XF analyzer.^29^ In the presence of glucose, glycolytic extracellular acidification rate was elevated in EC exposed to low compared with high shear stress (Figure 7D). In addition, glycolytic capacity, which is the ability of the glycolytic pathway to upregulate in time of energy need, was assessed after the addition of oligomycin that is a specific inhibitor of the mitochondrial ATP synthase. Glycolytic capacity was also higher in cells exposed to low shear stress compared with those cultured under high shear (Figure 7D). As a control, the glucose analogue 2 deoxyglucose (an inhibitor of glycolysis) was used to inhibit glycolysis and establish the nonglycolytic extracellular acidification rate. Collectively, these data indicate that low shear stress drives glycolysis via HIF1α-dependent induction of glycolytic enzymes.

### HIF1α-Dependent Glycolysis Enhanced Proliferation and Inflammation in EC Exposed to Low Shear Stress

**Figure 8.**
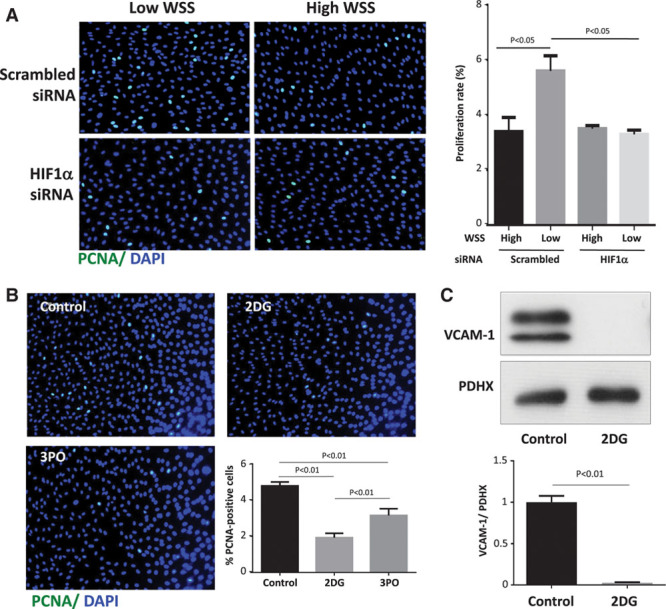
Low shear stress enhanced endothelial proliferation and inflammation via glycolysis. A, Human umbilical vein endothelial cells (HUVEC) were transfected with small interfering RNA (siRNA) targeting HIF1α (hypoxia-inducible factor 1α) or with scrambled sequences. After 24 h, cells were exposed to orbital flow to generate low (5 dyn/cm^2^) or high (11 dyn/cm^2^) wall shear stress (WSS). **B**, HUVEC were exposed to orbital flow to generate low WSS in the presence of 2 deoxyglucose (2DG; 5 mmol/L) or 3-(3-Pyridinyl)-1-(4-pyridinyl)-2-propen-1-one (3PO; 10 μmol/L) or dimethyl sulfoxide (DMSO) vehicle as a control. **A** and **B**, After 72 h, EC proliferation was quantified by immunofluorescent staining using anti-PCNA (proliferating cell nuclear antigen) antibodies and costaining using 4′,6-diamidino-2-phenylindole (DAPI). Representative images are shown. The % PCNA-positive cells were calculated for multiple fields of view. **C**, HUVEC were exposed to orbital flow to generate low WSS in the presence of 2DG (5 mmol/L) or DMSO vehicle for 72 h. They were exposed to tumor necrosis factor α (10 ng/mL) for the final 4 h. The expression levels of VCAM-1 and PDHX (pyruvate dehydrogenase complex component X) were assessed by Western blotting using specific antibodies. Representative blots are shown. Bands were quantified by densitometry. **A**–**C**, Data were pooled from 3 independent experiments, and mean expression±SEM is shown. Differences between means were analyzed using a 2-way (**A**) or 1-way (**B**) ANOVA with the Bonferroni correction for multiple pairwise comparisons or a paired *t* test (**C**).

We next investigated whether HIF1α-dependent glycolysis influences EC proliferation and inflammation because these processes are involved in the initiation of atherosclerosis at low shear regions.^2–10^ This was studied using cultured cells exposed to flow in vitro and 2 different measures of proliferation (PCNA [proliferating cell nuclear antigen] and Ki67 staining). Gene silencing of HIF1α significantly reduced proliferation of EC exposed to low shear but did not alter EC exposed to high shear conditions (Figure 8A; Figure X in the online-only Data Supplement), indicating that low shear stress induces EC proliferation via HIF1α. The contribution of enhanced glycolysis to proliferation was assessed using 2 deoxyglucose or 3-(3-Pyridinyl)-1-(4-pyridinyl)-2-propen-1-one (an inhibitor of PFKFB3^33^). Pretreatment of EC with either compound significantly reduced EC proliferation under low shear stress conditions (Figure 8B; Figure XI in the online-only Data Supplement). Moreover, the expression of inflammatory molecules in EC exposed to low shear stress was significantly reduced by silencing of Cezanne (Figure VIII in the online-only Data Supplement) or pretreatment of cells with 2DG (Figure 8C). Pretreatment of EC with 2DG did not alter the expression of Cezanne or HIF1α (Figure XII in the online-only Data Supplement), suggesting that glycolysis does not feedback to control Cezanne-HIF1α signaling. Collectively, these data indicate that low shear stress enhances EC proliferation and inflammatory activation via HIF1α-dependent induction of glycolysis enzymes.

### HIF1α Promotes EC Dysfunction at Low Shear Stress Regions of Arteries In Vivo

We validated our in vitro observations by imposing low shear stress in murine carotid arteries using the partial ligation model that promotes arterial wall remodeling and atherosclerosis in hypercholesterolemic mice.^25^ It should be noted that low shear stress is not sufficient to drive vascular inflammation per se, but it primes ECs for enhanced responses to inflammatory stimuli.^6^ Because atherosclerosis is a lipid-driven disease, we analyzed the function of HIF1α in partially ligated carotid arteries using hypercholesterolemic mice (ApoE^−/−^ mice exposed to a high-fat diet for 6 weeks). We previously demonstrated by morphometry analysis of cross-sections and microcomputed tomography that lesion area in partially ligated carotid arteries was reduced by ≈50% and that lumen volume was increased by ≈30% in HIF1α^EC-cKO^ ApoE^−/−^ mice compared with HIF1α^+/+^ ApoE^−/−^ mice.^23^ Correspondingly, it was demonstrated by morphometry of partially ligated carotid arteries that HIF1α^EC-cKO^ ApoE^−/−^ mice displayed a trend toward reduced total wall area compared with HIF1α^+/+^ ApoE^−/−^ mice (HIF1α^EC-cKO^: 39 660±9961 μm^2^; HIF1α^+/+^: 50 495±8423 μm^2^; mean±SEM; n=5; *P*<0.09), whereas medial thickness was unaltered by HIF1α deletion from EC (HIF1α^EC-cKO^: 26 854±3794 μm^2^; HIF1α^+/+^: 24 370±1097 μm^2^; *P*=0.19).

**Figure 9.**
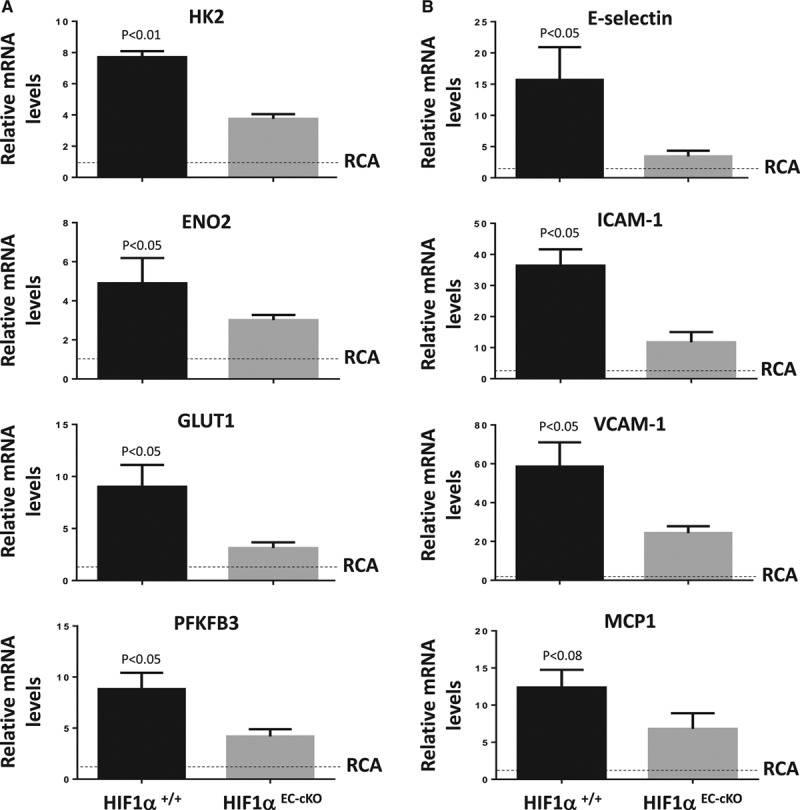
HIF1α (hypoxia-inducible factor 1α) induces glycolytic enzymes and inflammatory transcripts in arterial endothelium exposed to low shear in vivo. HIF1α^EC-cKO^ or HIF1α^+/+^ mice were subjected to partial ligation of the left carotid artery (LCA), whereas the right carotid artery (RCA) was sham-operated as a baseline control (RCA; unligated). After surgery, mice were exposed to a Western diet for 6 wk. The expressions of (**A**) regulators of glycolysis (hexokinase 2 [HK2], enolase 2 [ENO2], glucose transporter [GLUT] 1, 6-phosphofructo-2-kinase/fructose-2,6-biphosphatase 3 [PFKFB3]) or (**B**) inflammatory molecules (E-selectin, ICAM-1 [intercellular adhesion molecule 1], VCAM-1 [vascular cell adhesion molecule 1], MCP-1 [monocyte chemotactic protein 1]) were assessed by quantitative reverse transcription polymerase chain reaction. Data were pooled from n=4 mice per group. Mean gene expression±SEM in the LCA is presented. Differences between mean LCA values were analyzed using an unpaired *t* test. Because differences between LCA and RCA values were not tested statistically, we have presented RCA values as a baseline comparator (normalized to 1; dotted line).

**Figure 10.**
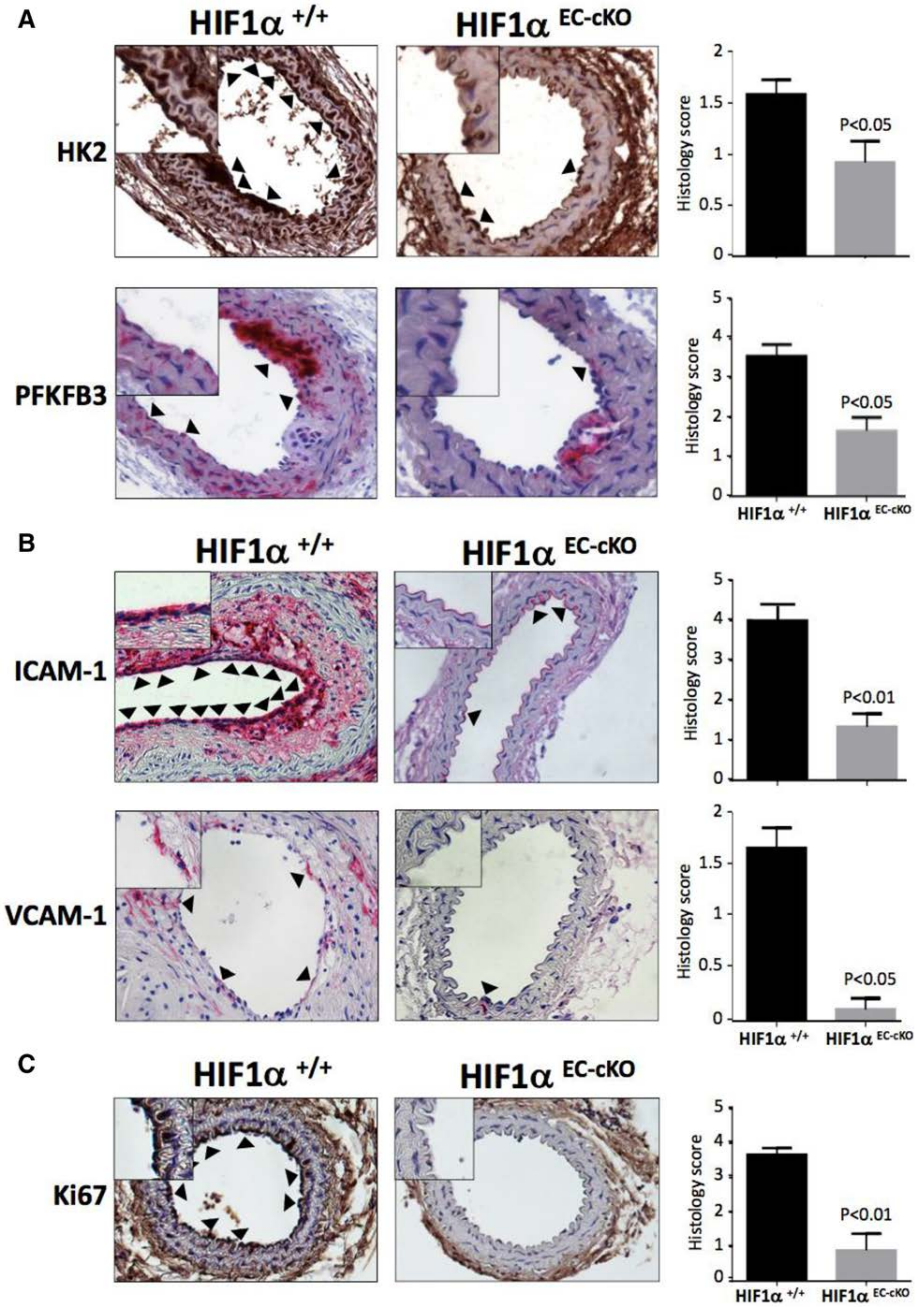
HIF1α (hypoxia-inducible factor 1α) induces glycolytic enzymes and inflammatory proteins in arterial endothelium exposed to low shear in vivo. HIF1α^EC-cKO^ or HIF1α^+/+^ mice were subjected to partial ligation of the left carotid artery. After surgery, mice were exposed to a Western diet for 6 wk. Immunostaining was performed to assess the expression of the glycolysis regulators hexokinase 2 (HK2) and 6-phosphofructo-2-kinase/fructose-2,6-biphosphatase 3 (PFKFB3; **A**), the inflammatory proteins ICAM-1 (intercellular adhesion molecule 1) and VCAM-1 (vascular cell adhesion molecule 1) (**B**), and the proliferation marker Ki67 (**C**) in cross-sections using DAB (3,3′-diaminobenzidine; brown) or NovaRed (red) substrates. n=5 mice per group were studied. Representative images are shown with arrowheads indicating EC that stained positive and high magnification insets. Histology scores of positive endothelial staining were pooled from 3 independent evaluations, and mean values±SEM are shown. Differences between means were analyzed using a Mann–Whitney test.

It was demonstrated by qRT-PCR (Figure 9A) and immunohistochemistry (Figure XIII in the online-only Data Supplement) that partially ligated left carotid arteries (LCA) of ApoE^−/−^ mice displayed enhanced expression of several regulators of glycolysis compared with sham right carotid arteries. The expression of inflammatory molecules was also enhanced in partially ligated LCA compared with sham-operated right carotid arteries (Figure 9B; Figure XIII in the online-only Data Supplement). The function of HIF1α in low shear-driven arterial inflammation and dysfunction was studied by inducible deletion of HIF1α from EC. First, it was demonstrated that the expression of HIF1α was significantly reduced in HIF1α^EC-cKO^ ApoE^−/−^ mice compared with HIF1α^+/+^ ApoE^−/−^ mice at both the mRNA and protein levels (Figure XIV in the online-only Data Supplement). It was subsequently demonstrated by qRT-PCR that the expression of glycolysis regulators (Figure 9A; HK2, enolase 2, glucose transporter 1, PFKFB3) and inflammatory molecules (Figure 9B; E-selectin, ICAM-1 [intercellular adhesion molecule 1], VCAM-1 [vascular cell adhesion molecule 1], MCP-1 [monocyte chemotactic protein 1]) in partially ligated LCA was significantly reduced in ApoE^−/−^ mice that lacked endothelial HIF1α (compare HIF1α^EC-cKO^ with HIF1α^+/+^). This concurred with immunostaining that demonstrated that the expression of proteins that regulate glycolysis (HK2, PFKFB3; Figure 10A) or inflammation (ICAM-1, VCAM-1; Figure 10B) was significantly reduced in partially ligated LCA by endothelial deletion of HIF1α. In parallel studies, we assessed the rate of EC proliferation in partially ligated LCA and demonstrated that this was significantly reduced by genetic deletion of HIF1α (Figure 10C). These data indicate that exposure of arteries to low shear stress leads to the activation of HIF1α driving glycolysis, inflammation, and EC proliferation.

In summary, mechanical shear stress induces NF-κB– and Cezanne-dependent upregulation of HIF1α at regions of arteries that are prone to atherosclerosis. HIF1α contributes to lesion initiation at atheroprone sites by activating glycolysis that enhances inflammation and EC proliferation. These data identify the Cezanne-HIF1α-glycolysis pathway as a novel mechanism contributing to lesion initiation at low shear stress regions of arteries.

## Discussion

Although HIF1α is canonically activated in response to hypoxia, here we show for the first time that mechanical low shear stress can activate HIF1α under normoxic conditions. The underlying molecular mechanism for low shear-induced HIF1α involves the transcription factor NF-κB that is known to be activated at low shear atheroprone regions.^7,8^ This finding is consistent with previous observations that NF-κB can induce transcription of the HIF1α gene in other contexts.^30,31,34,35^ We reasoned that a second mechanism should be involved because transcriptional activation of the HIF1α gene per se is unlikely to enhance protein levels in the face of PHD- and VHL-mediated degradations. We observed abundant expression of PHD and VHL in EC exposed to low shear stress, suggesting that HIF1α is hydroxylated and ubiquitinated in these conditions. We, therefore, analyzed the expression of Cezanne, a deubiquitinating enzyme^36^ that can rescue HIF1α from VHL-mediated degradation by cleaving ubiquitin from it.^32^ Cezanne was enhanced under low shear stress conditions in both in vitro and in vivo models, and manipulation of its activity or expression revealed that Cezanne enhances HIF1α expression at the protein level. Our collective data suggest a model where low shear stress enhances HIF1α via dual processes: NF-κB–dependent induction of HIF1α mRNA and subsequent Cezanne-dependent stabilization of HIF1α protein via ubiquitin editing. Our study also indicates that a functional interplay exists between EC mechanoresponses and hypoxia because exposure to low shear stress enhanced HIF1α activation in response to hypoxic signaling. Thus, EC at atheroprone sites are primed for responses to localized hypoxia, which may be caused by secondary flows that convect oxygen away from the arterial wall^37^ or from consumption of oxygen by metabolically active macrophages.^38^

The Cezanne protein was initially described by this laboratory as a negative regulator of NF-κB activation downstream from tumor necrosis factor or interleukin-1 receptor signaling.^27,39^ Thus, it was surprising to observe that Cezanne and NF-κB can co-operate to enhance HIF1α expression in low shear conditions. However, our subsequent studies revealed that silencing of Cezanne had modest effects on canonical NF-κB activation (RelA Ser536 phosphorylation) and did not alter noncanonical NF-κB (measured by p100 processing to p52) in EC exposed to low shear stress (data not shown). Thus, we suggest that cross-talk between Cezanne and NF-κB may differ according to physiological context; Cezanne can regulate NF-κB downstream from tumor necrosis factor α or interleukin-1 receptors but not in response to low shear stress. Consistent with this, tumor necrosis factor and interleukin-1 receptors signal to NF-κB via the generation of polyubiquitin chains that can be dismantled by Cezanne,^27,36^ whereas shear stress controls NF-κB via alternative pathways that may be insensitive to Cezanne.^8,40^ It is also interesting to compare our observations with those of Passerini et al^6^ who found using microarrays that Cezanne mRNA was enriched in the descending thoracic aorta (a high shear stress site) compared with the inner curvature of the aortic arch (low shear stress site). Further work is required to assess whether Cezanne regulation by shear stress differs between the aortic arch and thoracic descending aorta

Although it is well established that HIF1α is expressed in mature atherosclerotic plaques,^22^ its role in atherogenesis remains a topic of intense study. Recent work revealed that oral administration of a PHD2 inhibitor reduced lesion formation in mice by lowering serum cholesterol levels,^41^ indicating that systemic activation of HIF1α plays an atheroprotective role via modulation of lipid metabolism. However, conditional deletion or enforced expression of HIF1α in specific cell types revealed that the effects of HIF1α on atherogenesis are complex and vary according to cellular context. For example, expression of HIF1α in vascular smooth muscle cells^42^ or macrophages^43^ promoted inflammation in atherosclerosis, whereas HIF1α activity in CD11c-positive antigen-presenting cells protected arteries from lesion formation by reducing T-cell infiltrates.^44^ Of particular note, a recent study revealed that HIF1α expression in EC promoted lesion formation by enhancing expression of proinflammatory microRNA-19a.^23^ Here, we focused on the pathway that controls HIF1α expression in vascular endothelium and the downstream mechanisms that contribute to the initiation of atherosclerosis. Our studies of arteries from young mice and pigs revealed for the first time that HIF1α is expressed preferentially at low shear stress regions of the arterial tree that are predilection sites for atherosclerosis. Previous studies demonstrated that low shear stress promotes atherosclerosis initiation by inducing high rates of EC proliferation^2–4^ that increase the permeability of arteries to cholesterol-containing lipoproteins^10^ and by activating inflammatory pathways.^5–9^ Our findings illuminate the underlying molecular mechanism by demonstrating that HIF1α drives inflammation and proliferation in low shear stress conditions. Interestingly, genetic deletion of HIF1α did not completely restore the expression of glycolytic enzymes or inflammatory molecules to the baseline value. This implies that although HIF1α is required for the full induction of glycolytic enzymes and inflammatory molecules in response to low shear stress, other molecules also contribute to this response independently from HIF1α. Thus, therapeutic targeting of HIF1α would be expected to dampen inflammation of arteries but not prevent it completely. Several signaling molecules including p53^4^ and JNK1^3^ (c-jun n-terminal kinase) have been implicated in enhanced EC turnover at low shear stress sites, and future studies should determine whether these pathways cross-talk with HIF1α in atheroprone endothelium.

Our studies revealed that HIF1α enhances EC proliferation at low shear stress sites by inducing glycolytic enzymes to upregulate glycolysis. However, a recent study demonstrated that high shear stress reduces the rate of glycolysis in atheroprotected EC by activating the transcription factor KLF2 (Kruppel like factor 2) for transcriptional repression of PFKFB3.^45^ Thus, EC metabolism is regulated by high and low shear stress that trigger opposing signaling pathways; high shear reduces glycolysis via KLF2, whereas low shear enhances it via HIF1α. It is well established that HIF1α-dependent glycolysis plays an essential role during angiogenesis because it allows rapid ATP generation under hypoxic conditions and produces intermediates for macromolecule synthesis, thus promoting EC proliferation and migration.^19–21^ This pathway is important in tumor vascularization, and clinical trials are underway to test the ability of glycolysis inhibitors to treat cancer.^46^ Now, we show for the first time that this pathway is activated in adult arteries, specifically at atheroprone sites where it contributes to lesion initiation by promoting excessive EC proliferation and inflammation. It is likely that glycolysis also contributes to the progression of atherosclerosis, and it has recently been linked to inflammation in coronary artery disease.^47^

In summary, we demonstrate for the first time that HIF1α can be activated mechanically by low shear stress. This noncanonical pathway, which requires activation of NF-κB and Cezanne, promotes HIF1α accumulation at atheroprone sites leading to excessive EC proliferation and focal inflammation. Thus, the mechanically activated Cezanne-HIF1α axis contributes to the initiation of lesions at branches and bends and may provide a novel therapeutic target to promote vascular function.

**Highlights**

- HIF1α (hypoxia-inducible factor 1α) can be activated by mechanical shearing of arterial endothelial cells in the presence of oxygen, a noncanonical mechanism that promotes HIF1α accumulation at atheroprone sites.
- The underlying mechanism involves dual processes; nuclear factor-κB–dependent induction of HIF1α mRNA and stabilization of HIF1α protein by the ubiquitin-editing enzyme Cezanne.
- HIF1α promotes atherogenic processes at low shear stress regions by inducing excessive endothelial proliferation and inflammation via upregulation of glycolysis.
- Thus, noncanonical mechanical activation of HIF1α plays an important role in focal endothelial dysfunction and has the potential to be targeted therapeutically to enhance vascular function.

## Acknowledgments

We thank Fiona Wright (University of Sheffield) for technical support.

## Sources of Funding

S. Feng, N. Bowden, V. Ridger, and P.C. Evans are funded by the British Heart Foundation (RG/13/1/30042). M. Fragiadaki is funded by Kidney Research UK. S. Allen is funded by the Motor Neurone Disease Association. H. Jo’s work was supported, in part, by funding from National Institutes of Health grants HL095070 and John and Jan Portman Professorship.

## Disclosures

None.

## Supplementary Material
