# Supplementary figures and images for: Mechanical Activation of Hypoxia-Inducible Factor 1α Drives Endothelial Dysfunction at Atheroprone Sites
Source: Arterioscler Thromb Vasc Biol. 2017 Oct 25;37(11):2087–101. doi: 10.1161/ATVBAHA.117.309249 (PMC5659306; doi:10.1161/ATVBAHA.117.309249)

# GRAPHICAL ABSTRACT

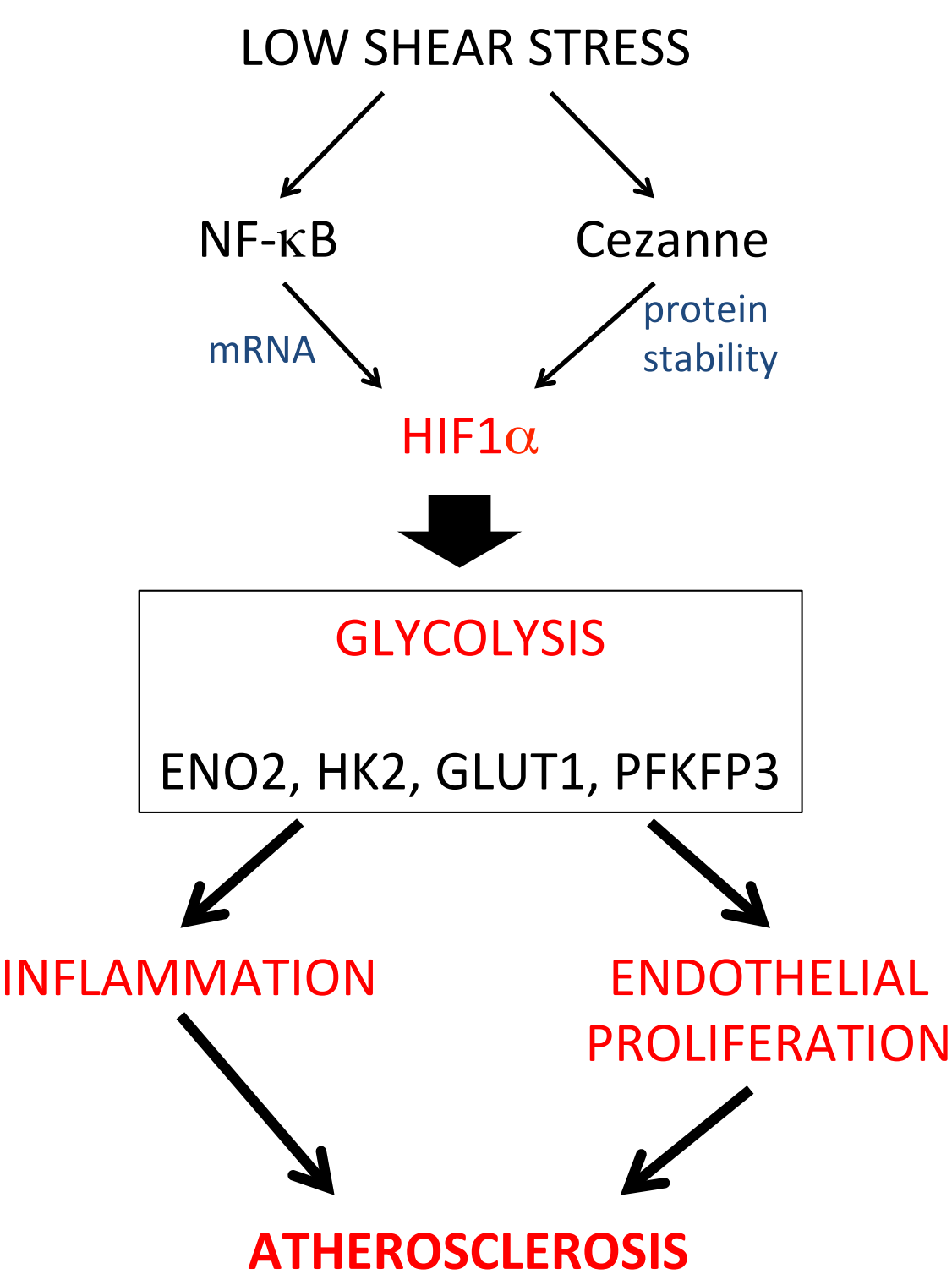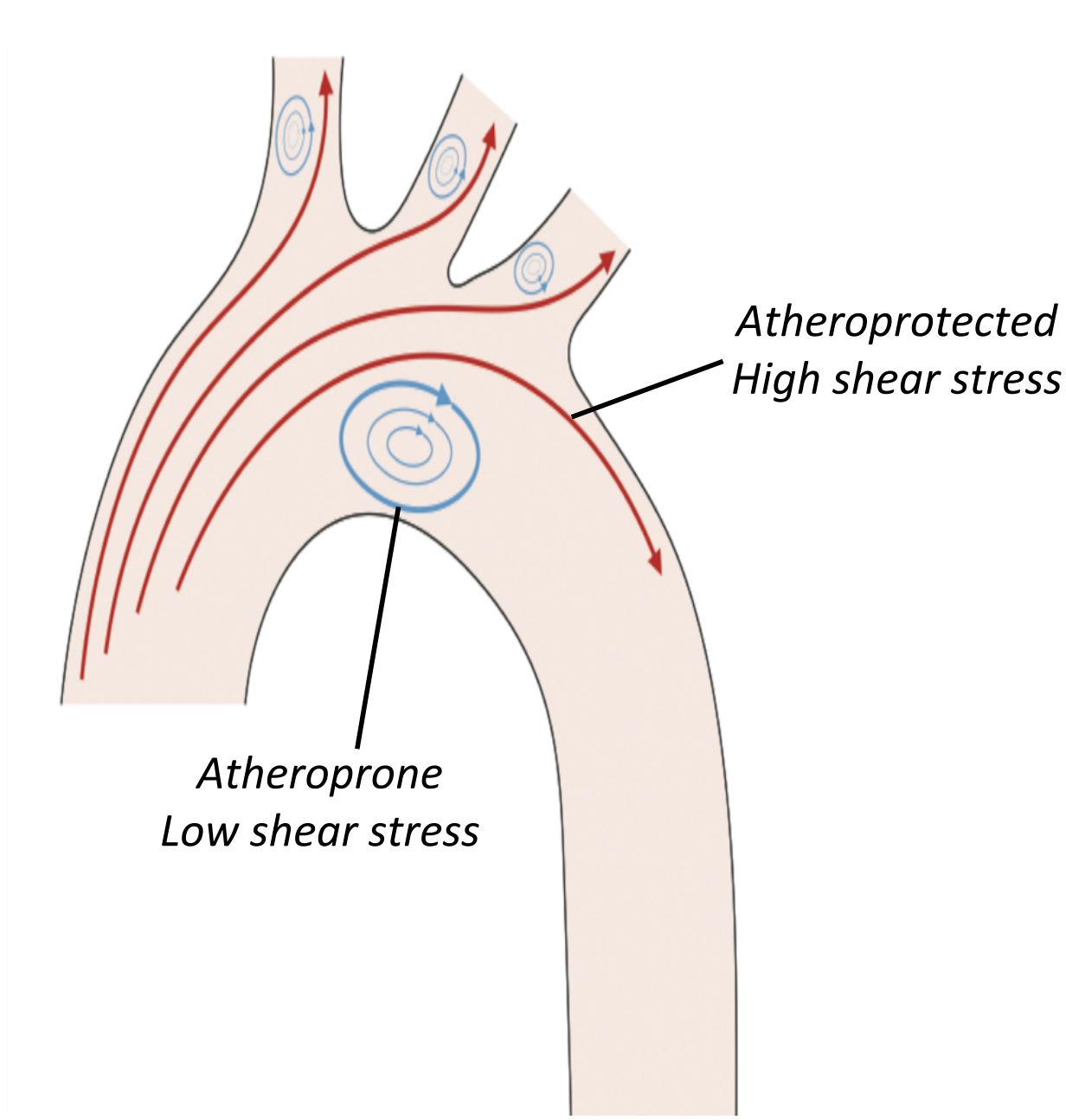

Supplement: Supplementary file 1 [file atv-37-2087-s001.pdf]
