# Supplementary material for: Mechanical Activation of Hypoxia-Inducible Factor 1α Drives Endothelial Dysfunction at Atheroprone Sites
Source: Arterioscler Thromb Vasc Biol. 2017 Oct 25;37(11):2087–101. doi: 10.1161/ATVBAHA.117.309249 (PMC5659306; doi:10.1161/ATVBAHA.117.309249)
Supplement: Supplementary file 2 [file atv-37-2087-s002.pdf]

MATERIALS AND METHODS

Isolation of EC from porcine aortae. Pig aortas from 4-6 month old animals (weight approximately 80kg) were obtained immediately after slaughter from a local abattoir. They were cut longitudinally along the outer curvature to expose the lumen. EC exposed to high (outer curvature) or low (inner curvature) wall shear stress (WSS) were harvested using collagenase (1 mg/ml for 10 minutes at room temperature) prior to gentle scraping.

Mouse lines. Mice were housed under specific-pathogen free conditions. All animal experiments were reviewed and approved by the local authorities in accordance with German animal protection law. HIF1α was deleted from endothelial cells (EC) of $ApoE^{-/-}$ mice by crossing $Hif1\alpha^{flox/flox}$ $ApoE^{-/-}$ mice VE-Cad-Cre-ER^{T2} mice which express Tamoxifen-activated Cre under control of the VE-cadherin promoter as described[1]. Experiments were carried out using female mice because vascular physiology varies between sexes in mice with females having more pronounced and less variable pathophysiological features[2]. Thus female VE-Cad-Cre-ER^{T2}/ $Hif1\alpha^{flox/flox}$/ $ApoE^{-/-}$ conditional knockout mice (called $HIF1\alpha^{EC-cKO}$) and VE-Cad-Cre-ER^{T2}/ $Hif1\alpha^{+/+}$/ $ApoE^{-/-}$ ($HIF1\alpha^{+/+}$) mice were treated with tamoxifen (2 mg/20 g body weight) dissolved in neutral oil via intraperitoneal (i.p.) injections for 5 consecutive days. After one week, mice aged 6-8 weeks were anesthetized (ketamine (80 mg/kg, i.p.), xylazine (10 mg/kg, i.p.)) and the left carotid artery was partially ligated by closing the external, internal and the occipital artery restricting blood outflow to the superior thyroid artery only as described[3]. The right carotid artery was sham-operated as a control. Mice were fed a high fat diet (0.15% cholesterol) for 6 weeks.

Staining of murine endothelium. The expression levels of specific proteins were assessed in EC at regions of the inner curvature (susceptible site) and outer curvature (protected site) of murine aortae by *en face* staining. Animals were killed by I.P injection of pentobarbital or by isofluorane overdose. Aortae were perfused *in situ* with PBS (at a pressure of approximately 100 mm Hg) and then perfusion-fixed with 4% Paraformaldehyde prior to harvesting. Fixed aortae were tested by immunostaining using specific primary antibodies and Alexafluor568-conjugated secondary antibodies (red). EC were identified by co-staining using anti-CD31 antibodies conjugated to the fluorophore FITC (green). Nuclei were identified using a DNA-binding probe with far-red emission (To-Pro-3). Stained vessels were mounted prior to visualization of endothelial surfaces *en face* using confocal laser-scanning microscopy (Zeiss LSM510 NLO inverted microscope). Isotype-matched monoclonal antibodies raised against irrelevant antigens or pre-immune rabbit sera were used as experimental controls for specific staining. The expression of particular proteins at each site was assessed by quantification of fluorescence intensity for multiple cells (at least 50 per site) using Image J (1.49p) and calculation of mean fluorescence intensities with standard error of the mean.

Consecutive sections at an interval of 120 μm made from experimental carotid arteries were immunostained using specific primary antibodies. Non-specific IgG served as a negative control. Primary antibodies were detected using horse radish peroxidase-conjugated secondary antibodies and 3,3-diaminobenzidine (DAB; brown) or NovaRed (red) substrates (Vector Laboratories, Burlingame, CA, USA).

Sections were counterstained using haematoxylin and eosin. The extent of EC staining in cross-sections was scored by 3 researchers blinded to the experimental conditions. Lesions were identified in carotid arteries by elastic van Gieson staining of serial sections (4–5 µm thick) at an interval of 100 µm, and the total vessel area and medial area were calculated by morphometry using Image J software.

EC culture and exposure to WSS. HUVEC were isolated using collagenase digestion. Cells were cultured on 1% gelatin and maintained in M199 growth medium supplemented with foetal bovine serum (20%), L-glutamine (4 mmol/L), endothelial cell growth supplement (30 µg/ml), penicillin (100 U/ml), streptomycin (100 µg/ml) and heparin (10 IU/ml). Human coronary artery EC (HCAEC) were purchased from PromoCell and cultured according to the manufacturer's recommendations. EC at passage 3-5 were cultured until confluent in 6 well plates and exposed to flow using an orbital shaking platform (PSU-10i; Grant Instruments) housed inside a cell culture incubator. The radius of orbit of the orbital shaker was 10 mm and the rotation rate was set to 210 rpm. This motion caused swirling of the culture medium over the cell surface generating low WSS (approximately 5 dynes/cm^2) with varied directionality at the centre and high (approximately 11 dynes/cm^2) WSS with uniform direction at the periphery. Alternatively, HUVEC were cultured on Ibidi® gelatin-coated µ-Slides (Ibidi GmbH) until they reached confluency. Flowing medium was then applied using the Ibidi® pump system to generate low (4 dyn/cm^2), low oscillatory (+/- 4 dyn/cm^2, 0.5 Hz) or high (13 dyn/cm^2) WSS. The slides and pump apparatus were enclosed in a cell culture incubator warmed to 37°C.

Gene silencing and overexpression. Cell cultures were transfected with siRNA sequences that are known to silence HIF1α (Dharmacon SMARTpool: ON-TARGETplus L-004018-00-0005, Dharmacon Individual: ON-TARGETplus J-004018-08-0002), RelA (Dharmacon SMARTpool: ON-TARGETplus L-003533-00-0005) or Cezanne (Dharmacon siGENOME D-008670-03) using the Lipofectamine® RNAiMAX transfection system (13778-150, Invitrogen) following the manufacturer's instructions. Alternatively, cells were transfected with the Neon™ Transfection system (Invitrogen; 1200 volts, 40 ms, 1 pulse). Final siRNA concentration used was 25nM for lipofectamine[4] and 50 mm by electroporation. After knockdown, cells were then incubated in complete M199 growth medium for 2 h before exposure to flow. Non-targeting scrambled sequences were used as a control (D-001810-01-50 ON-TARGETplus Non targeting siRNA#1, Dharmacon). HUVEC were transfected with expression vectors containing IκBα (pCMV-IκBα, GFP-Cezanne or GFP-Cezanne Cys/Ser (catalytically inactive)[5] using Lipofectamine (ThermoFisher).

Comparative real time PCR. RNA was extracted using the RNeasy Mini Kit (74104, Qiagen) and reverse transcribed into cDNA using the iScript cDNA synthesis kit (1708891, Bio-Rad). The levels of human, porcine or murine transcripts were assessed using quantitative real time PCR (qRT-PCR) using gene-specific primers (Supplementary Table 1). Reactions were prepared using SsoAdvanced universal SYBR®Green supermix (172-5271, Bio-rad) and following the manufacturer's instructions, and were performed in triplicate. Relative gene expression was calculated by comparing the number of thermal cycles that were

necessary to generate threshold amounts of product. Fold changes were calculated using the ΔΔCt method. Data were pooled from at least three independent experiments and mean values were calculated with SEM.

Immunoprecipitation of HIF1α. Cells were lysed using ice-cold lysis buffer (50 mM Tris (pH 7.4), 250 mM NaCl, 0.3% Triton X-100, 1mM EDTA, 10μM MG132) supplemented with protease inhibitor cocktail (Roche, Basel, Switzerland). Lysates were subjected to three freeze-thaw cycles before immunoprecipitation using anti-HIF1α antibodies. Beads were then washed extensively using lysis buffer.

Western blotting Total cell lysates were isolated using lysis buffer (containing 2% SDS, 10% Glycerol and 5% β-mercaptoethanol). Western blotting was carried out using specific antibodies against HIF1α, HIF2α, Cezanne, IκBα, HK2, ENO2, PFKFB3, VCAM-1, PDHX, Calnexin (Supplementary Table 2) and horse radish peroxidase-conjugated secondary antibodies obtained commercially from Dako and chemiluminescent detection was carried out using ECL Prime® (GE Healthcare).

Chromatin immunoprecipitation. A commercial kit was used (Cell Signaling Technology). HUVECs were fixed using formaldehyde and nuclei were purified and subjected to sonication. For immunoprecipitation reactions, the nuclear lysates were incubated overnight at 4°C with protein G magnetic beads coated with either anti-RelA (sc-372; Santa Cruz) or with anti-histone H3 antibodies or isotype-matched IgG control (both from Cell Signaling Technology). Immunocomplexes were washed, and co-precipitating DNA fragments were quantified by real-time PCR using specific DNA primers (Supplementary Table 1).

Immunofluorescent staining of cultured EC. The expression levels of proteins were assessed by immunostaining using specific antibodies followed by widefield fluorescence microscopy (LeicaDMI4000B). HUVEC were fixed with Paraformaldehyde (4%) and permeabilised with Triton X-100 (0.1%). Following blocking with goat serum for 30 min monolayers were incubated for 16 h with primary antibodies against HIF1α, PCNA (proliferation marker) or Ki67 (proliferation marker) and AlexaFluor488- or Alexafluor568-conjugated secondary antibodies. Nuclei were identified using the DNA-binding probe DAPI (Sigma). Image analysis was performed using Image J software (1.49p) to calculate average fluorescence. Isotype controls or omission of the primary antibody was used to control for non-specific staining.

Glycolysis assay. HUVEC were plated in a Cell Tak-coated Seahorse cell culture plate in XF assay media pH 7.4 (Seahorse Bioscience) supplemented with 2 mM glutamine and 25 mM glucose. Extracellular acidification rate (ECAR) was measured basally prior to addition of oligomycin to assess glycolytic capacity. After reading basic ECAR and maximum ECAR induced by oligomycin, glycolysis was suppressed via addition of 2 deoxyglucose (2DG) thus allowing measurement of non-glycolytic ECAR as described[6]. Glycolytic ECAR was calculated by taking the ECAR value prior to oligomycin injection and subtracting the ECAR value in the presence of 2DG. Glycolytic capacity was calculated by subtracting ECAR in the presence of oligomycin from ECAR in the presence of 2DG.

Statistics. Differences between samples were analysed using an unpaired or paired Student's t-test or ANOVA with the Bonferonni correction for multiple pairwise comparisons.

Supplementary Table 1 PCR primers.

Mouse	Forward 5'-3'	Reverse 5'-3'
Mouse HK2	TCGCATATGATCGCCTGCTTA	TCTGAGAGACGCATGTGGTAG
Mouse ENO2	GAGAAGATTTGGGCCCGAGAG	CGGAAAAGACCTTTGGCAGT
MouseGLUT1	GGATCACTGCAGTTCGGCTA	AGCGGTGGTTCCATGTTTGA
Mouse PFKFB3	GAAGCTGACTCGCTACCTCA	TGTACTGCTTCACAGCCTCA
Mouse MCP1	CTGGAGCATCCACGTGTTGG	CAGCTTCTTTGGGACACCTG
Mouse ICAM1	CCCTGGTCACCGTTGTGAT	GAACAGTTCACCTGCACGGAC
Mouse VCAM1	TGGGAGCCTCAACGGTACTT	AGCAATCGTTTTGTATTCAGGGG
Mouse E-selectin	GAATGCCTCGCGCTTTCTCT	GAGCTCACTGGAGGCATTGTA
**Pig**	**Forward 5'-3'**	**Reverse 5'-3'**
Pig HIF1α	TGGTACTCACAGATGATGGTGAC	CATTTCCTCATGGTCGCACG
Pig HK2	GAACACGGAGAGTTCCTGGC	CCTTCTGGAGTCCGTTGTCT
Pig ENO2	GGCCTCTCTGTGGTGGAAC	ATTGGCCCCAAACTTGGACT
Pig GLUT1	GGCATCAACGCGGTTTTCTAT	AGACTCACCGACACGACAGT
Pig GLUT3	TGCTCCTGAGGCGATCATAAA	GACCACAGGGACGTGAGTAA
Pig PFKFB3	CTGACCCGCTACCTCAACTG	AGGAGCTGTACTGCTTGACG
**HUMAN**	**Forward 5'-3'**	**Reverse 5'-3'**
Human HIF1α	TGGTTCTCACAGATGATGGTGAC	CTCATTTCCTCATGGTCACATGG
Human HK2	TCACGGAGCTCAACCATGAC	GCTCCAAGCCCTTTCTCCAT
Human ENO2	CCACAGTGGAGGTGGATCTCT	TCATAGATGCCCGTAGAGGCT
Human GLUT1	TCCCTGCAGTTTGGCTACAA	CAGGATGCTCTCCCCATAGC
Human GLUT3	CTCTTCGTCAACCGCTTTGG	CCCATAAAGCAGCCACCAGT
Human PFKFB3	CCGTTGGAACTGACGCAGA	CACAGGATCTGGGCAACGAG
Human Cezanne	GAGTTGAGAAGGAAGCGTT	CACTCCTTCTGCCATTCATC
Human HIF1A promoter	CTTCGGAGAAGGCGCAGAGT	GTGCTCGTCTGTGTTTAGCG

Supplementary Table 2 Antibodies: suppliers and concentrations used.

Antibody	Company	Use	Dilution Ratio	Final Concentration
HIF1α	Mouse monoclonal Anti-Human HIF-1α, 610959, BD Biosciences	WB	1/500	5μg/ml
HIF1α	Rabbit monoclonal antibody to HIF1α, ab51608, Abcam	IF	1/100	10μg/ml
VCAM1	Rabbit monoclonal anti-VCAM1 antibody, Ab134047, Abcam	WB	1/5000	80ng/ml
		ICC	1/200	2μg/ml
PCNA	Rabbit polyclonal anti- PCNA antibody, Ab18197, Abcam	IF	1/100	2μg/ml
PFKFB3	Rabbit monoclonal anti- PFKFB3 antibody, Ab181861, Abcam	WB	1/3000	0.2μg/ml
		ICC	1/100	5μg/ml
ENO2	Rabbit polyclonal NSE/ENO2 Antibody [NB100-91898], Novus	WB	1/2000	0.5μg/ml
HK2	Rabbit polyclonal Hexokinase II Antibody [NBP2-16814], Novus	WB	1/5000	0.2μg/ml
		IF	1/200	5μg/ml
HIF2α	Abcam, Rabbit polyclonal anti-HIF-2-alpha antibody, Ab199	WB	1/1000	1μg/ml
ICAM1	Rabbit polyclonal anti-ICAM1 antibody, 10020-I-AP, ProteinTech	ICC	1/250	5μg/ml
PDHX	Rabbit polyclonal E3BP(PDHX) antibody, sc-98751, Santa Cruz	WB	1/5000	40ng/ml
Calnexin	Mouse monoclonal Anti-calnexin, 610524, BD Biosciences	WB	1/5000	50ng/ml
Cezanne	Rabbit polyclonal to OTUD7B, 16605-I-AP, ProteinTech	WB	1/3000	50ng/ml
		IF	1/100	2.5μg/ml
Ki67	Rabbit polyclonal to Ki67, ab15580, Abcam	IF	1/100	5μg/ml

WB, Western blotting; IF, immunofluorescence; ICC, immunocytochemistry.

REFERENCES

1. Akhtar S, Hartmann P, Karshovska E, Rinderknecht F-A, Subramanian P, Gremse F, Grommes J, Jacobs M, Kiessling F, Weber C, Steffens S, Schober A. Endothelial hypoxia-inducible factor-1 alpha promotes atherosclerosis and monocyte recruitment by upregulating microrna-19a. *Hypertension*. 2015;66:1220-1226.

2. Caligiuri G, Nicoletti A, Zhou X, Tornberg I, Hansson GK. Effects of sex and age on atherosclerosis and autoimmunity in apoE-deficient mice. *Atherosclerosis*. 1999;145:301-308.

3. Nam D, Ni C-W, Rezvan A, Suo J, Budzyn K, Llanos A, Harrison D, Giddens D, Jo H. Partial carotid ligation is a model of acutely induced disturbed flow, leading to rapid endothelial dysfunction and atherosclerosis. *Am J Physiol-Heart Circ Physiol*. 2009;297:H1535-H1543.

4. Zografou S, Basagiannis D, Papafotika A, Shirakawa R, Horiuchi H, Auerbach D, Fukuda M, Christoforidis S. A complete rab screening reveals novel insights in weibel-palade body exocytosis. *J Cell Sci*. 2012;125:4780-4790.

5. Enesa K, Zakkar M, Chaudhury H, Luong L, Rawlinson L, Mason JC, Haskard DO, Dean JLE, Evans PC. Nf-kappab suppression by the deubiquitinating enzyme cezanne: A novel negative feedback loop in pro-inflammatory signaling. *J Biol Chem*. 2008;283:7036-7045.

6. Richardson K, Allen SP, Mortiboys H, Grierson AJ, Wharton SB, Ince PG, Shaw PJ, Heath PR. The effect of sod1 mutation on cellular bioenergetic profile and viability in response to oxidative stress and influence of mutation-type. *Plos One*. 2013;8.
