# Supplementary material for: Mechanical Activation of Hypoxia-Inducible Factor 1α Drives Endothelial Dysfunction at Atheroprone Sites
Source: Arterioscler Thromb Vasc Biol. 2017 Oct 25;37(11):2087–101. doi: 10.1161/ATVBAHA.117.309249 (PMC5659306; doi:10.1161/ATVBAHA.117.309249)
Supplement: Supplementary file 3 [file atv-37-2087-s003.pdf]

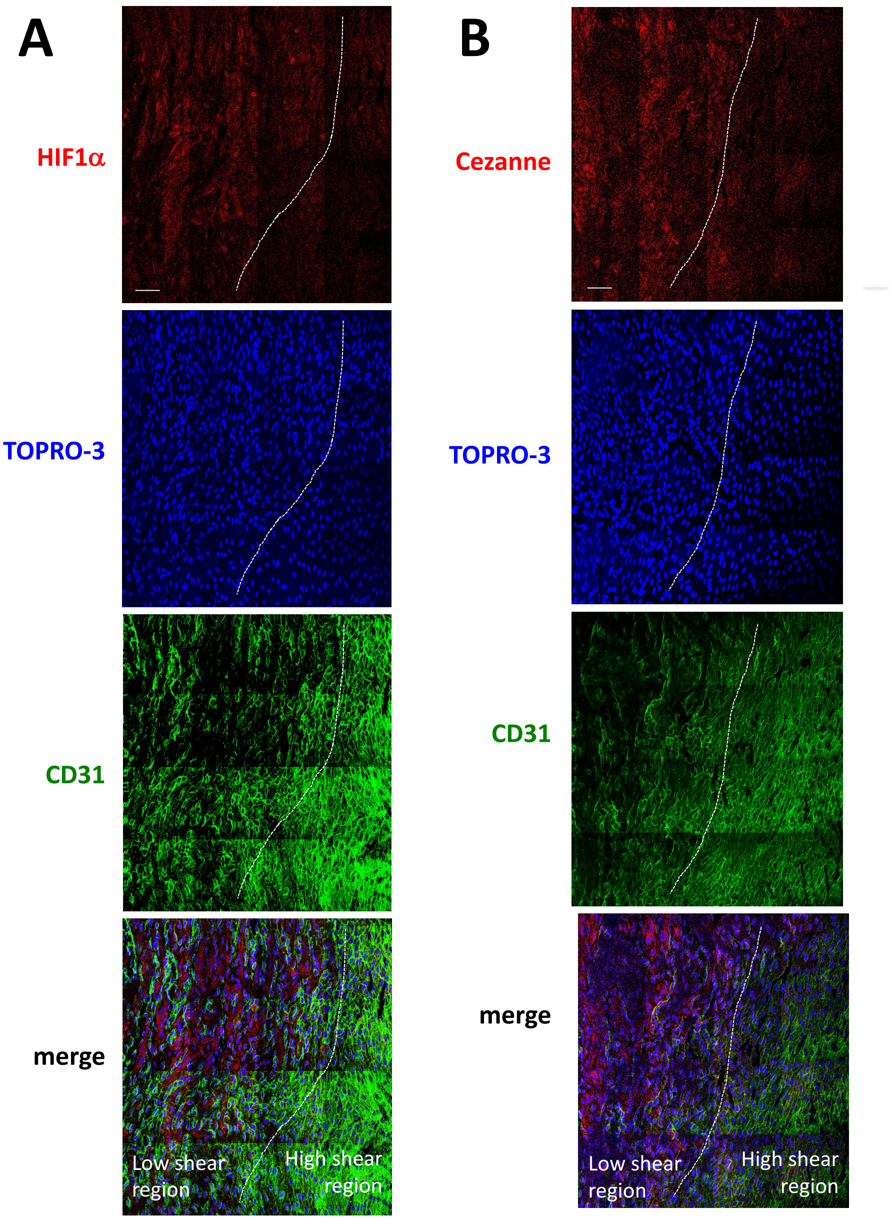

Supplementary Figure I. Low magnification images of en face staining of HIF1α and Cezanne in the murine aorta.

The expression of HIF1α (A) and Cezanne (B) was assessed at low shear stress (inner curvature) and high shear stress (outer curvature) regions of the murine aorta by *en face* staining. EC were identified by co-staining with anti-CD31 antibodies conjugated to FITC (green). Cell nuclei were identified using TOPRO3 (blue). Regions exposed to high and low shear stress were identified by anatomical landmarks and confirmed by assessment of nuclei which are aligned specifically under high shear stress. Images representing tiling of multiple fields of view are presented. Note the distinct difference in HIF1α and Cezanne expression between high and low shear stress regions (delineated by a broken white line). Scale bar, 20 μm.

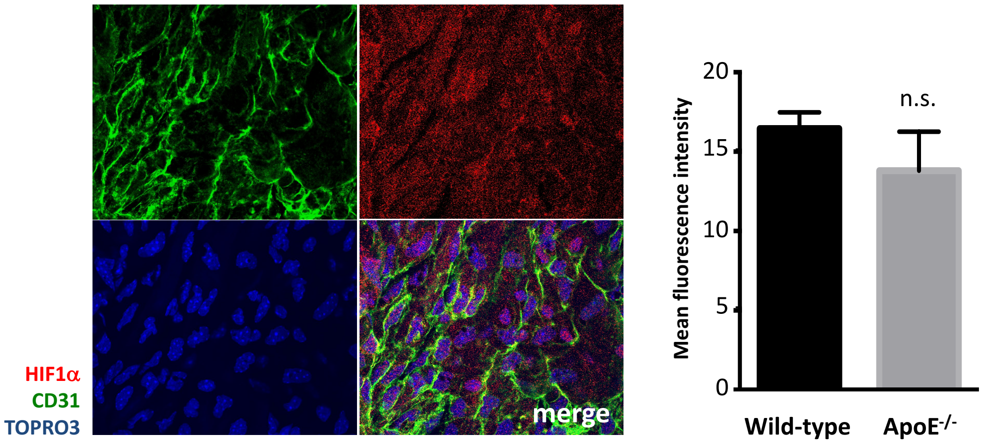

Supplementary Figure II. HIF1α is expressed by endothelial cells overlying plaques at low shear stress regions.
ApoE$^{-/-}$ mice aged 8 weeks were exposed to a high fat diet for 6 weeks (n=4). C57BL/6 (wild-type) mice aged 14 weeks were also studied (n=4). The expression of HIF1α was quantified at a low wall shear stress region (inner curvature) by en face staining. EC were identified by co-staining with anti-CD31 antibodies conjugated to FITC (green). Cell nuclei were identified using TOPRO3 (blue). A representative image of HIF1α expression in EC overlying a plaque in the ApoE$^{-/-}$ model is shown (left). Mean levels of HIF1α expression (+/- SEM) were calculated for ApoE$^{-/-}$ and wild-type mice (right). Differences between means were analysed using a paired t-test. n.s., not significant.

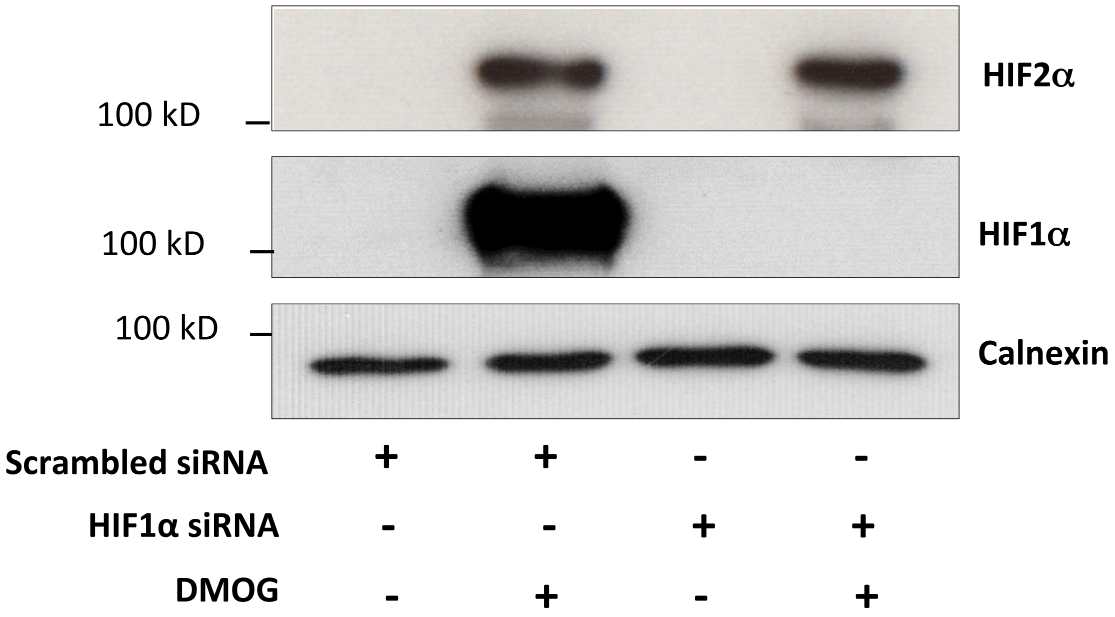

Supplementary Figure III. Validation of HIF1α siRNA.
HUVEC were transfected with siRNA targeting HIF1α or with scrambled sequences. After 24 h, cells were exposed to DMOG for 4 h. The expression levels of HIF1α and HIF2α were assessed by Western blotting using specific antibodies, and anti-Calnexin antibodies were used to control for total protein levels. Representative blots are shown.

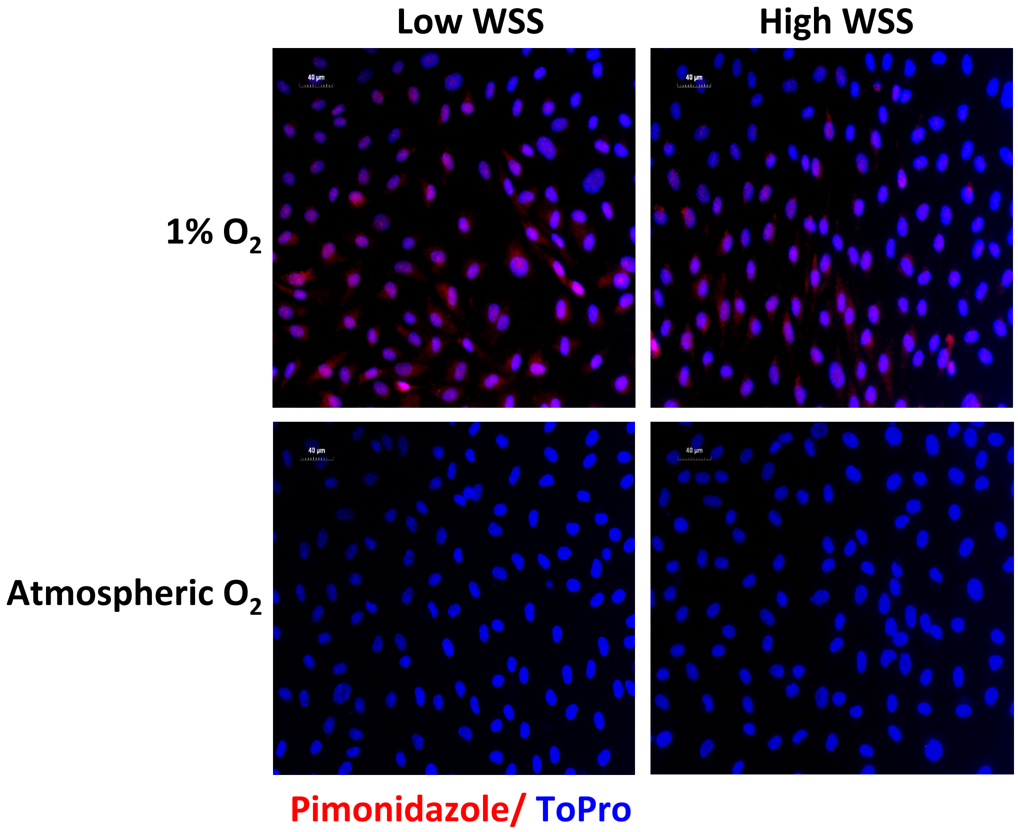

Supplementary Figure IV. Endothelial cells exposed to low shear stress in atmospheric oxygen were not hypoxic.

HUVEC were exposed to orbital flow to generate low (5 dyn/cm^2) or high (11 dyn/cm^2) wall shear stress (WSS) either within a hypoxic chamber (1% O_2) or under atmospheric levels O_2. After 72 h, the cells were treated with Pimonidazole (60 ng/ml) for 90 min before fixation with 4% PFA and staining using rabbit polyclonal anti-pimonidazole antibodies. Hypoxic cells were assessed by Pimonidazole staining (red). Cell nuclei were identified using DAPI (blue).

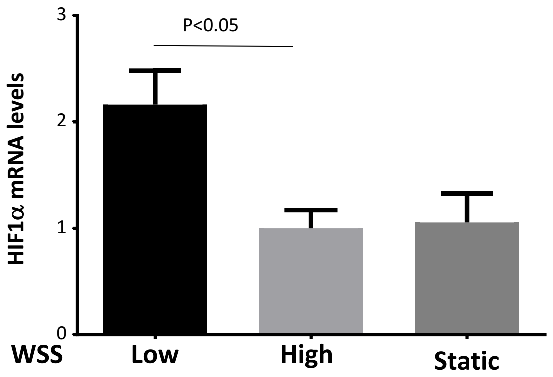

Supplementary Figure V. Low shear stress induced HIF1a mRNA in coronary artery EC.

Human coronary artery EC were exposed to low (5 dyn/cm^2) or high (11 dyn/cm^2) wall shear stress (WSS) for 72 h using an orbital system or were exposed to static conditions. HIF1α mRNA was quantified by qRT-PCR. Data were pooled from three different donors and mean HIF1α expression levels +/- SEM are shown. Differences between means were analysed using a 1-way ANOVA with the Bonferroni correction for multiple pairwise comparisons.

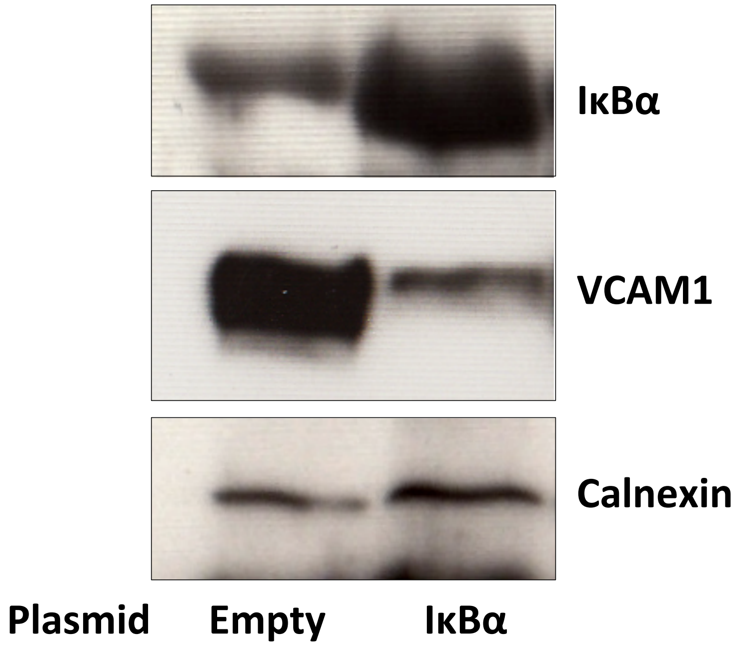

Supplementary Figure VI. Validation of enforced IκBα expression.
HUVEC were transfected with pCMV-IκBα to inhibit NF-κB or with an empty plasmid as a control. After 24 h, the expression levels of IκBα and VCAM-1 were assessed by Western blotting using specific antibodies, and anti-Calnexin antibodies were used to control for total protein levels. Representative blots are shown.

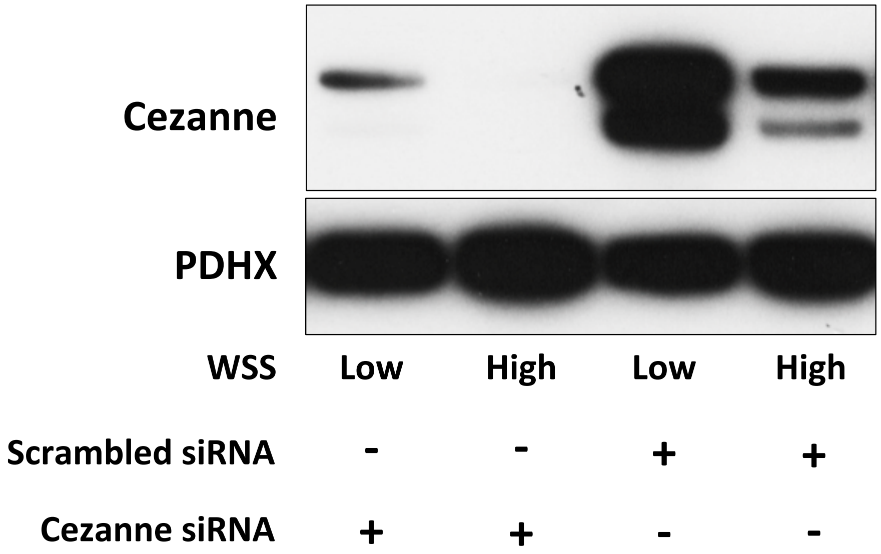

Supplementary Figure VII. Validation of antibodies used to detect Cezanne by Western blotting.
HUVEC were transfected with siRNA targeting Cezanne or with scrambled sequences. Cells were then exposed to orbital flow to generate low WSS (5 dyn/cm^2). After 72 h, the expression levels of Cezanne were assessed by Western blotting using specific antibodies, and anti-PDHX antibodies were used to control for total protein levels. Representative blots are shown.

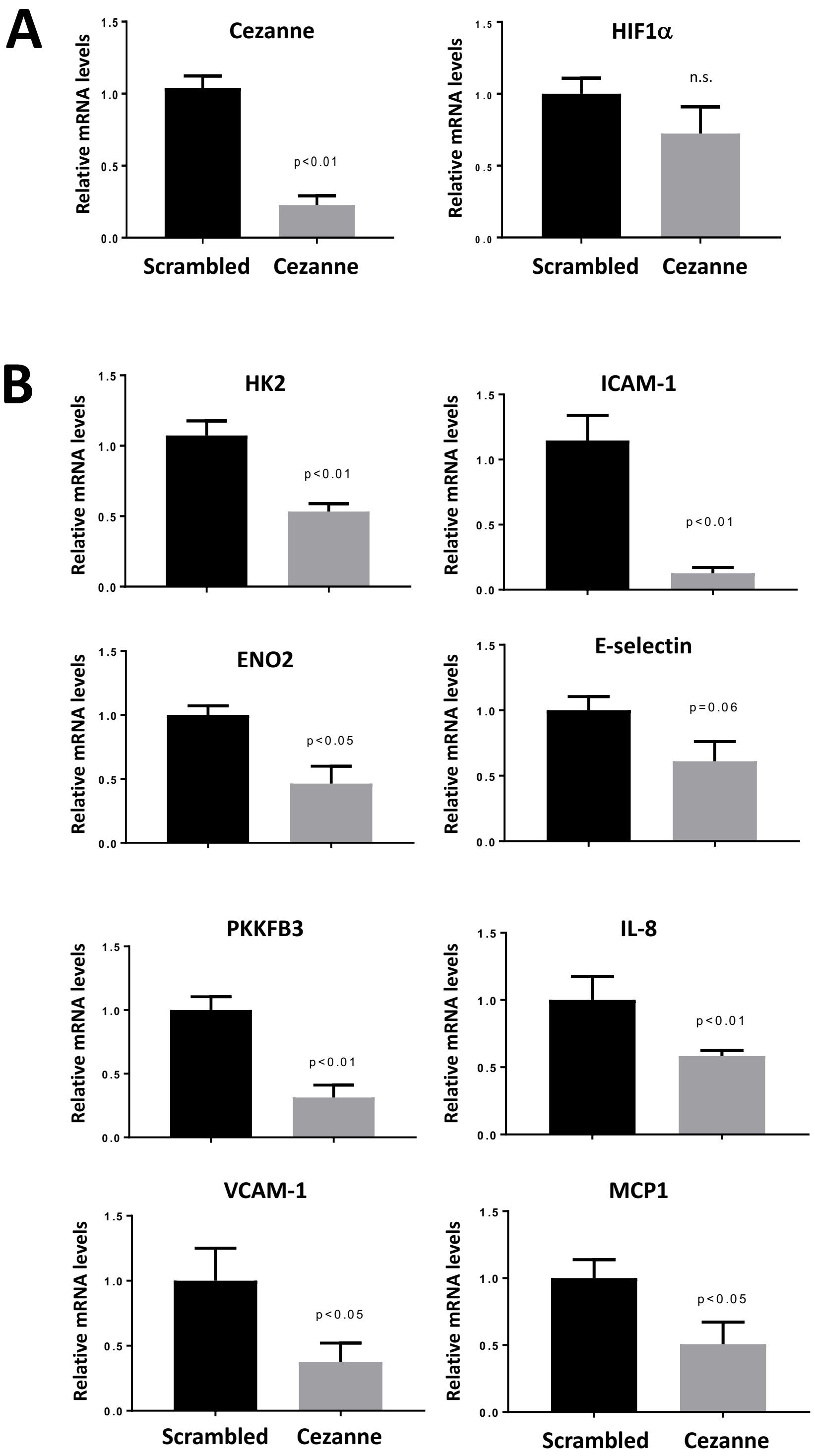

Supplementary Figure VIII. Cezanne positively regulates the expression of glycolysis genes and inflammatory molecules in EC exposed to low shear stress.

HUVEC were transfected with siRNA targeting Cezanne or with scrambled sequences. Cells were subsequently exposed to orbital flow to generate low WSS (5 dyn/cm^2) for 72 h. The expression levels of Cezanne, HIF1α (A) or HK2, ENO2, PFKFB3, VCAM-1, ICAM-1, E-selectin, IL-8 and MCP1 (B) mRNA were assessed by qRT-PCR. Data were generated from three independent experiments and differences between means were analysed using a paired t-test.

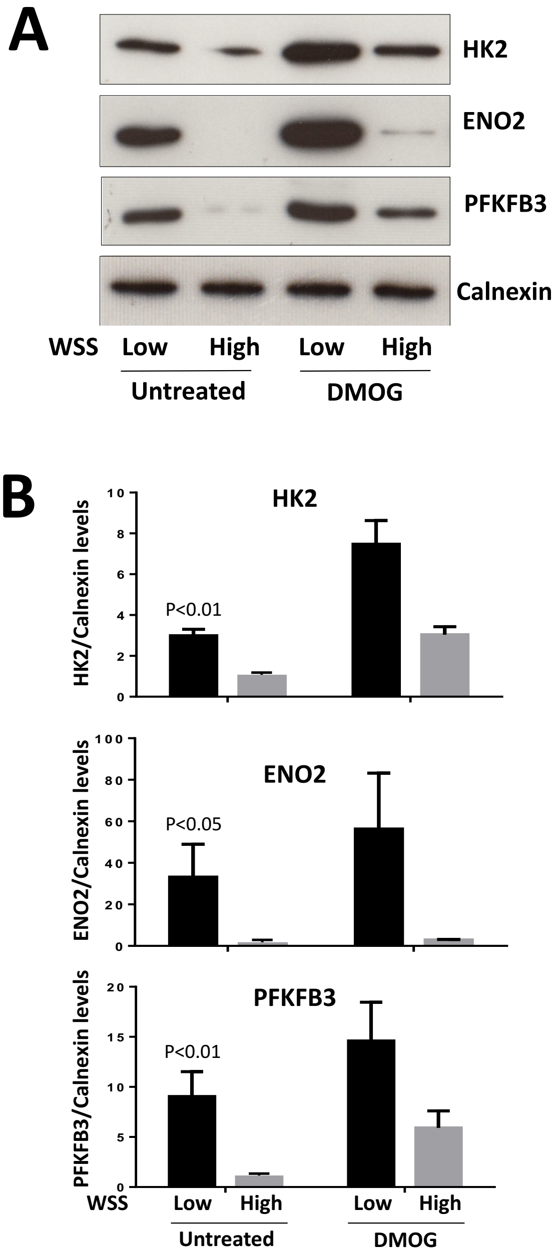

Supplementary Figure IX. Low shear stress primed EC for hypoxic signaling.

HUVEC were exposed to orbital flow to generate low (5 dyn/cm^2) or high (11 dyn/cm^2) wall shear stress (WSS). After 72h, cells were treated with DMOG for 4 h. The expression levels of HK2, ENO2 and PFKFB3 were assessed by Western blotting using specific antibodies, and anti-Calnexin antibodies were used to control for total protein levels. (A) Representative blots are shown. (B) Bands were quantified by densitometry. Data were pooled from three independent experiments and mean expression +/- SEM is shown. Differences between means were analysed using a 2-way ANOVA with the Bonferroni correction for multiple pairwise comparisons.

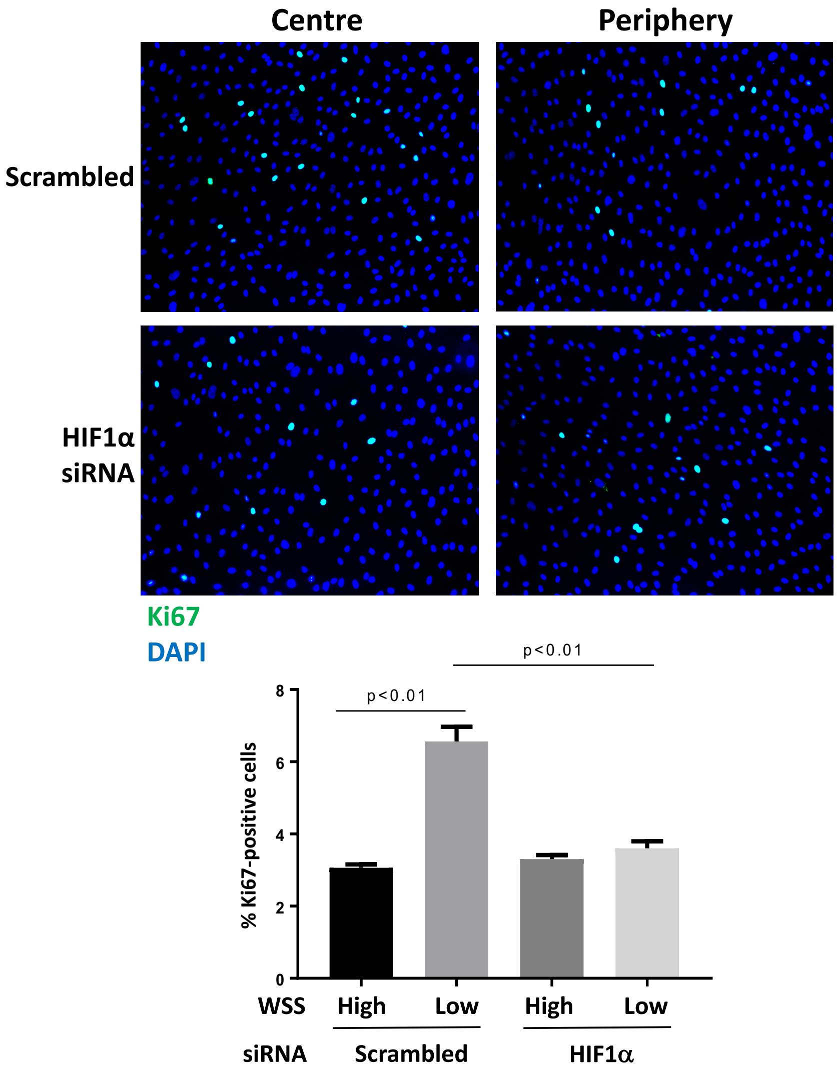

Supplementary Figure X. Low shear stress enhanced endothelial proliferation (Ki67 staining) via HIF1α.

HUVEC were transfected with siRNA targeting HIF1α or with scrambled sequences. After 24 h, cells were exposed to orbital flow to generate low (5 dyn/cm^2) or high (5 dyn/cm^2) shear stress. After 72 h, EC proliferation was quantified by immunofluorescent staining using anti-Ki67 antibodies and co-staining using DAPI. Representative images are shown. The % Ki67-positive cells were calculated for multiple fields of view. Data were pooled from three independent experiments and mean expression +/- SEM is shown. Differences between means were analysed using a 2-way ANOVA with the Bonferroni correction for multiple pairwise comparisons.

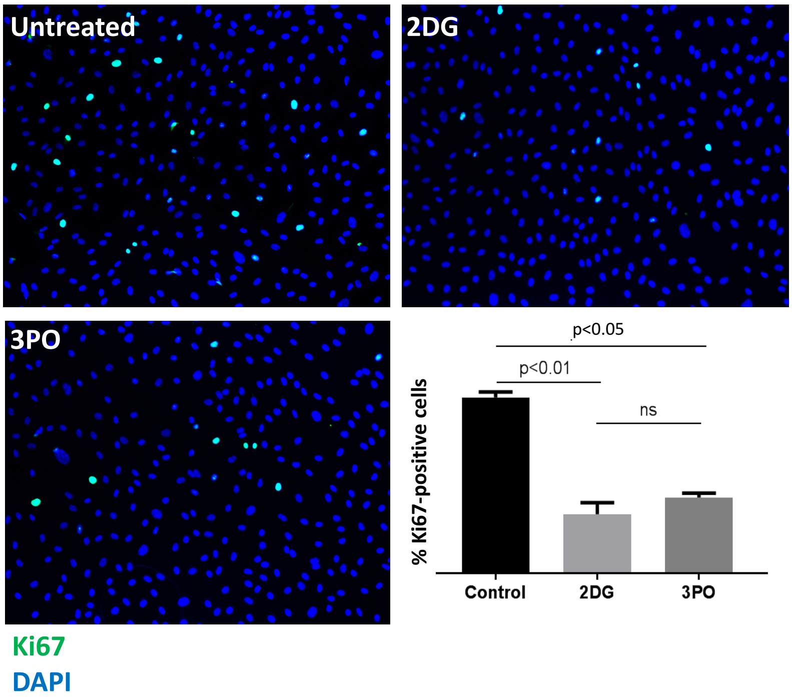

Supplementary Figure XI. Low shear stress enhanced endothelial proliferation (Ki67 staining) via glycolysis.
HUVEC were exposed to orbital flow to generate low shear stress (5 dyn/cm^2) in the presence of 2DG (5 mM) or 3PO (10 μM) or DMSO vehicle as a control. After 72 h, EC proliferation was quantified by immunofluorescent staining using anti-Ki67 antibodies and co-staining using DAPI. Representative images are shown. The % Ki67-positive cells were calculated for multiple fields of view. Data were pooled from three independent experiments and mean expression +/- SEM is shown. Differences between means were analysed using a 1-way ANOVA with the Bonferroni correction for multiple pairwise comparisons.

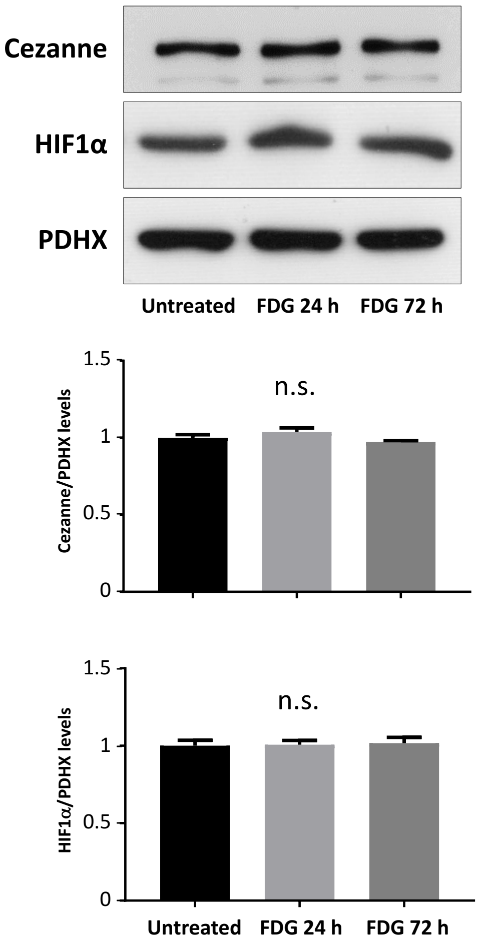

Supplementary Figure XII. FDG treatment did not alter the expression of Cezanne or HIF1α in EC.

HUVEC were exposed to FDG for 24 h or 72 h or remained untreated as a control. The expression levels of HIF1α and Cezanne were assessed by Western blotting using specific antibodies, and anti-PDHX antibodies were used to control for total protein levels. Representative blots are shown. Bands were quantified by densitometry. Data were pooled from three independent experiments and mean levels +/- SEM are shown. Differences between means were analysed using 1-way ANOVA with the Bonferroni correction for multiple pairwise comparisons. n.s., not significant.

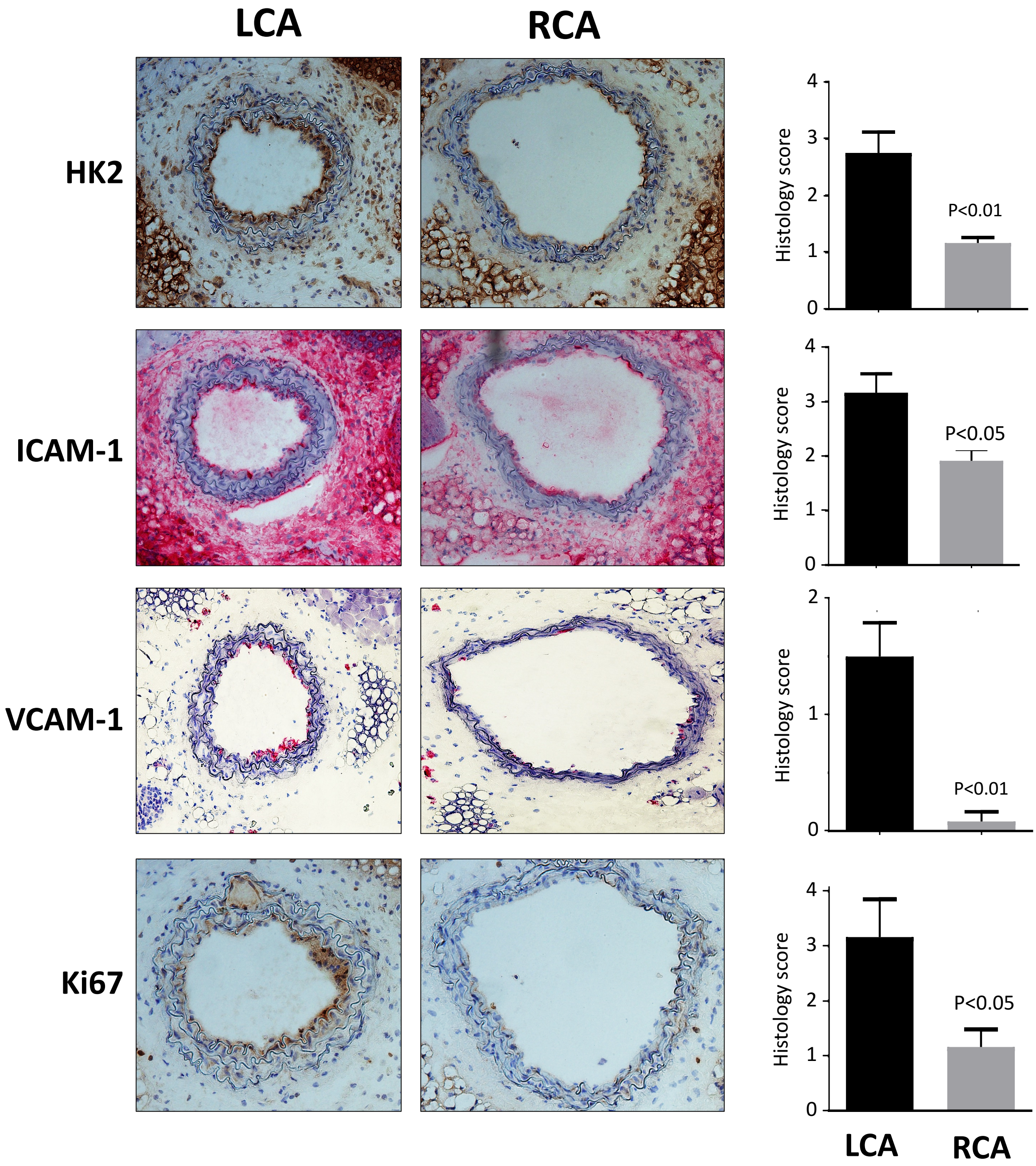

Supplementary Figure XIII. Glycolytic enzymes, inflammatory proteins and proliferation were induced by low shear in vivo. ApoE$^{-/-}$ mice were subjected to partial ligation of the left carotid artery (LCA). After surgery, mice were exposed to a Western diet for 6 weeks. Immunostaining was performed to assess the expression of the glycolysis regulator HK2, the inflammatory proteins ICAM-1 and VCAM-1, and the proliferation marker Ki67 in cross-sections using DAB (brown) or NovaRed (red) substrates. n=4 mice per group were studied. Representative images are shown. Histology scores of positive endothelial staining were pooled from 3 independent evaluations and mean values +/- SEM are shown. Differences between means were analysed using a Mann Whitney test.

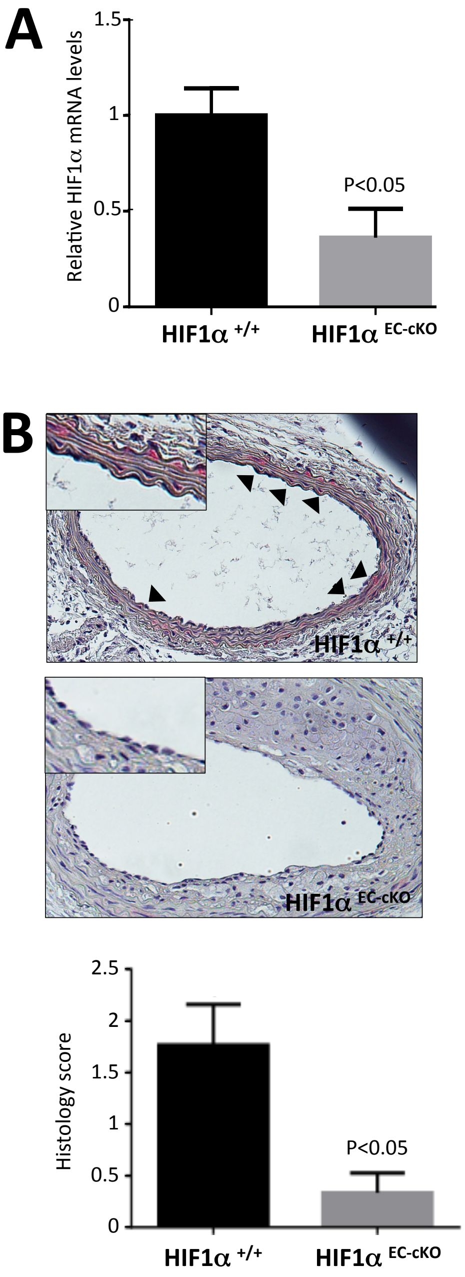

Supplementary Figure XIV. Validation of inducible HIF1α genetic deletion from EC.

HIF1α$^{EC\text{-}cKO}$ or HIF1α$^{+/+}$ mice were subjected to partial ligation of the left carotid artery. After surgery, mice were exposed to a Western diet for 6 weeks. (A) The expression of HIF1α was assessed by qRT-PCR. Data were pooled from n=4 mice per group. Differences between means were analysed using an unpaired t-test. (B) Immunostaining was performed to assess the expression of HIF1α protein in cross-sections using NovaRed (red) substrate. n=5 mice per group were studied. Representative images are shown with arrowheads indicating EC that stained positive and high magnification insets. Histology scores of positive endothelial staining were pooled and mean values +/- SEM are shown. Differences between means were analysed using a Mann Whitney test.
